# Rearrangement-mediated cis-regulatory alterations in advanced patient tumors reveal interactions with therapy

**DOI:** 10.1016/j.celrep.2021.110023

**Published:** 2021-11-16

**Authors:** Yiqun Zhang, Fengju Chen, Erin Pleasance, Laura Williamson, Cameron J. Grisdale, Emma Titmuss, Janessa Laskin, Steven J.M. Jones, Isidro Cortes-Ciriano, Marco A. Marra, Chad J. Creighton

**Affiliations:** 1 Division of Biostatistics, Dan L. Duncan Comprehensive Cancer Center, Baylor College of Medicine, Houston, TX, USA; 2 Canada’s Michael Smith Genome Sciences Centre at BC Cancer, Vancouver, British Columbia, Canada; 3 Department of Medical Oncology, BC Cancer, Vancouver, British Columbia, Canada; 4 Department of Medical Genetics, University of British Columbia, Vancouver, British Columbia, Canada; 5 Department of Molecular Biology and Biochemistry, Simon Fraser University, Vancouver, British Columbia, Canada; 6 European Molecular Biology Laboratory, European Bioinformatics Institute, Hinxton, Cambridge, CB10 1SD, UK; 7 Department of Medicine, Baylor College of Medicine, Houston, TX, USA; 8 These authors contributed equally; 9 Lead contact

## Abstract

The global impact of somatic structural variants (SVs) on gene regulation in advanced tumors with complex treatment histories has been mostly uncharacterized. Here, using whole-genome and RNA sequencing from 570 recurrent or metastatic tumors, we report the altered expression of hundreds of genes in association with nearby SV breakpoints, including oncogenes and G-protein-coupled receptor-related genes such as *PLEKHG2*. A significant fraction of genes with SV-expression associations correlate with worse patient survival in primary and advanced cancers, including *SRD5A1*. In many instances, SV-expression associations involve retrotransposons being translocated near genes. High overall SV burden is associated with treatment with DNA alkylating agents or taxanes and altered expression of metabolism-associated genes. SV-expression associations within tumors from topoisomerase I inhibitor-treated patients include chromatin-related genes. Within anthracycline-treated tumors, SV breakpoints near chromosome 1p genes include *PDE4B*. Patient treatment and history can help understand the widespread SV-mediated cis-regulatory alterations found in cancer.

## In brief

By analyzing 570 advanced cancers, Zhang et al. reveal that structural variation patterns of altered cis-regulation, in terms of well-established oncogenes and tumor suppressor genes, correlate with enriched gene classes, patient survival, retrotransposon and enhancer hijacking, and prebiopsy therapy. Therapy associations may represent tumor subtype selection or mechanisms of resistance.

## Graphical Abstract

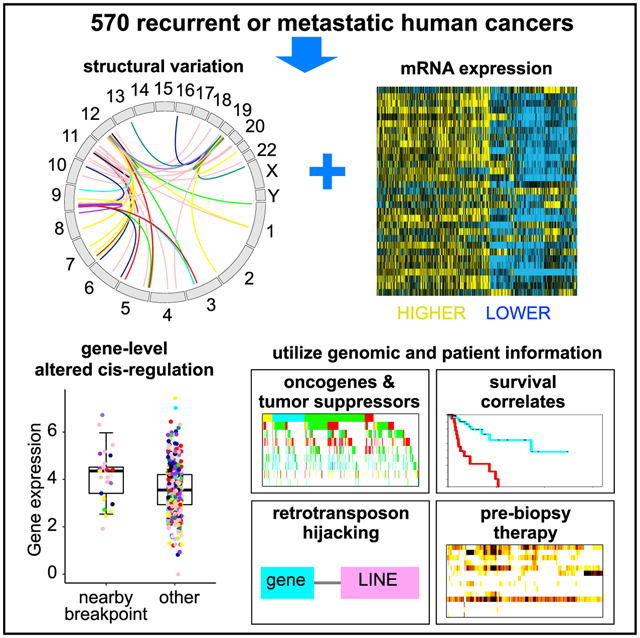

## INTRODUCTION

While ~1% of the human genome represents protein-coding regions, or exons, some 80% of DNA is estimated to have biochemical functions—including gene regulation—outside of the well-studied protein-coding regions (ENCODE Project Consortium, 2012). In cancer, somatic DNA alterations extend beyond gene boundaries and impact gene regulation, with one well-known example involving single nucleotide variants (SNVs) in the *TERT* promoter region, causing overexpression of the gene (Heidenreich et al., 2014). Structural variants (SVs)—whereby the broken ends of DNA from two different locations rejoin to form a new chromosomal arrangement—represent another class of DNA alteration impacting gene cis-regulation. Well-known mechanisms involved in SV-mediated altered regulation include enhancer hijacking (Peifer et al., 2015) and disruption of topologically associating domains (TADs) (Weischenfeldt et al., 2017), although other mechanisms could exist. A recent important milestone in the study of noncoding alterations in cancer was led by the Pan-Cancer Analysis of Whole Genomes (PCAWG) Consortium (ICGC-TCGA Pan-Cancer Analysis of Whole Genomes Network, 2020). While “hotspot” or recurrent somatic SV breakpoint patterns appear less common in the PCAWG cohort of primary cancers (Rheinbay et al., 2020), for hundreds of genes the nearby presence of an SV breakpoint recurrently associates with altered expression (Zhang et al., 2020). This phenomenon of widespread rearrangement-mediated cis-regulatory alterations has been confirmed in pediatric brain tumors (Zhang et al., 2021b) and cancer cell lines (Zhang et al., 2021a).

As part of the Personalized OncoGenomics (POG) Program at BC Cancer, 570 advanced and metastatic cancers from patients treated at a tertiary care center (known as the POG570 cohort) were profiled using whole-genome sequencing (WGS) and RNA sequencing (RNA-seq) (Pleasance et al., 2020). In contrast, the PCAWG studies are mainly representative of pretreated primary cancer, while the POG570 cohort represents tumors collected after patient therapy, with most patients (n = 466, 82%) receiving systemic therapy before biopsy. Significant associations with patient therapy or survival involving SNV events, overall SNV burden, and mutational signatures were identified. In the POG570 cohort, recurrent noncoding SNV events were found in regulatory region hotspots of genes, including *TERT*, *PLEKHS1*, *AP2A1*, and *ADGRG6*. The initial POG570 study focused mainly on SNVs and insertion-deletions (indels) involving coding and noncoding regions but did not consider SV-mediated cis-regulatory alterations.

This present study utilizes the POG570 datasets to analyze high-coverage WGS and RNA-seq data from 570 advanced and metastatic cancers. By integrating somatic SV calls with gene expression data, we observe a widespread impact of SVs on the regulation of genes in the vicinity of SV breakpoints, with a large portion of SV-associated genes in the POG570 cohort not being observed in PCAWG or other patient cohorts. A significant fraction of genes with SV-expression associations in the POG570 cohort was associated with worse patient survival or therapy. Among other things, SV-expression associations could involve retrotransposons being translocated near genes. Chromothripsis and high overall SV burden was associated with treatment with DNA-damaging agents or mitotic inhibitors and with altered expression of metabolism-associated genes. Gene-level SV-expression associations were also defined within POG570 subgroups defined by patient therapy. For example, SV-expression associations within tumors from topoisomerase I inhibitor-treated patients included chromatin-related genes, and SV breakpoints within anthracycline-treated tumors included *PDE4B*.

## RESULTS

### Somatic SV-associated cis-regulatory alterations in advanced cancers

**Figure 1.**
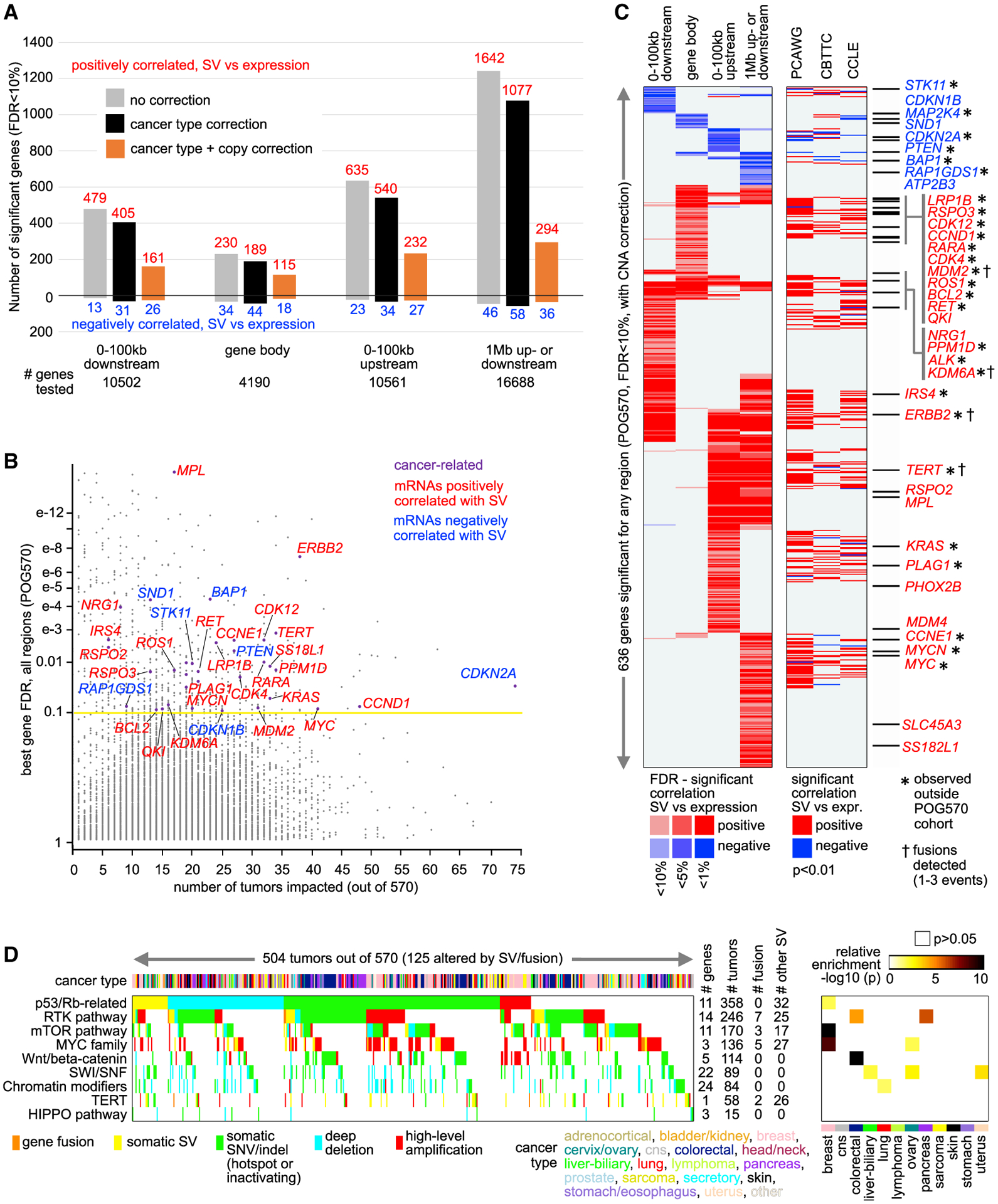
Genes with altered expression associated with nearby somatic SV breakpoints in the POG570 cohort (A) For each of the indicated genomic region windows examined, numbers of significant genes (FDR < 10%) show a correlation between expression and associated SV breakpoint event. Numbers above and below the zero point of the y axis denote positively and negatively correlated genes, respectively. SVExpress evaluated significant associations when correcting for cancer type (black) and for both cancer type and gene-level CNA (orange). (B) Significance of genes in the POG570 cohort, as plotted (y axis) versus the number of patients impacted (expression > 0.4 SD from sample median) by a nearby SV breakpoint [for any region examined in (A), correcting for both cancer type and CNA]. “Cancer-related,” by COSMIC. (C) Heatmap of significance patterns for 636 genes significant for any region window (FDR < 10%, correcting for both cancer type and CNA). Shown alongside the POG570 results are the corresponding gene-level results from other sample cohorts. Red denotes a significant positive correlation; blue, significant negative correlation. Genes listed are cancer associated by COSMIC. (D) Pathway-centric view of somatic alterations (representing 504 patients with at least one somatic alteration in the indicated pathways), involving key cancer-related pathways and genes (see STAR Methods). The right panel represents the significance of enrichment (one-sided Fisher’s exact test) of gene alteration events for each pathway within the given cancer type versus the rest of the tumors (focusing on the 12 most represented cancer types). See also Figures S1 and S2 and Tables S1, S2, and S3.

We assessed gene-level associations between expression and nearby somatic SV breakpoints across the POG570 cohort (Table S1), composed of advanced and metastatic tumors from patients treated in a tertiary cancer clinic and representing 25 histologies with biopsies from 18 organ groups (Pleasance et al., 2020). Some 68,554 somatic SVs were detected across the POG570 cohort, ranging from 3 to 813 SVs detected per sample. By integrating RNA-seq with WGS-derived SV data using SVExpress (Zhang et al., 2021a), hundreds of genes showed altered gene expression in relation to nearby SV breakpoints, including breakpoints located either downstream or upstream of genes and breakpoints occurring in the gene body (Figure 1A; Table S2). Incorporating statistical corrections for gene-level copy number alteration (CNA) decreased the overall numbers of significant genes, consistent with previous findings of widespread association of SV breakpoints with copy gain (Zhang et al., 2018). The remaining significant genes after CNA correction represent those for which SV-associated copy gain or loss alone would not entirely explain the observed SV-associated altered expression across samples. Many more genes showed positive correlations with SV breakpoints (i.e., expression was higher when a nearby SV breakpoint was present) than negative correlations. This observation again was in line with previous studies (Zhang et al., 2018, 2019, 2020, 2021a, 2021b). Genes positively correlated with SV breakpoints included many known oncogenes, while genes negatively correlated included many known tumor suppressor genes (Figures 1B and 1C).

At a false discovery rate (FDR) of < 10%, correcting for both cancer histological type and CNA, a set of 636 genes (of 16,720 total) was identified across the set of region windows examined relative to genes: 100 kb upstream, 100 kb downstream, within the gene, and 1 Mb upstream or downstream (Figures 1C and S1A). The 1-Mb region window—whereby the integration method weighted the relative gene distances of the breakpoints (Zhang et al., 2019), giving the most weight to breakpoints closest to the gene—could capture the long-range effects of SVs, including enhancer hijacking (Harewood and Fraser, 2014). A small fraction of the SV events underlying the top 636 genes involved gene fusions, as evidenced by concurrent SV and chimeric transcript detection. The top 545 genes involving overexpression represented just 12 fusion events involving eight genes. Alternatively, within-gene breakpoints involving underexpression would represent gene disruption by SVs. The 636 genes were enriched (p < 0.01, one-sided Fisher’s exact test) for cancer-associated genes by the Catalogue of Somatic Mutations in Cancer (COSMIC) (Forbes et al., 2017) (Figure 1C). Genes with significant SV-expression associations tended to be closer in distance to each other (Figure S1B), as SVs falling near a gene cluster could conceivably disrupt multiple genes together. We compared the top SV-expression associations from the POG570 cohort with results from three other cohorts with combined WGS and RNA-seq data (Table S3): the PCAWG cohort (1220 patients) (Zhang et al., 2020), the Children’s Brain Tumor Tissue Consortium (CBTTC) cohort (759 patients and 894 pediatric brain tumors) (Zhang et al., 2021b), and the Cancer Cell Line Encyclopedia (CCLE) (Ghandi et al., 2019; Zhang et al., 2021a). We observed a high degree of overlap between the POG570 results and results from the other cohorts examined (Figure 1C), while for 344 of the 636 POG570 top genes, there was no association (p < 0.01) in the other cohorts. For the POG570 cohort, we could also identify gene-level SV-expression associations by cancer type (Figures S1C–S1F).

### SVs involved with pathway-level alterations and G-protein-coupled receptors

We found somatic SVs to considerably extend the numbers of advanced cancers somatically altered for critical pathways. Taking a set of cancer-associated pathways and related genes previously annotated based on domain knowledge (Chen et al., 2017a, 2018), we examined the POG570 tumors for alteration in these pathways (Table S1). Alterations considered were gene fusion, SV-mediated altered cis-regulation or gene disruption, SNV or indel, and deep deletion or amplification. By tumor and pathway, we conservatively considered SVs as a last measure, after no other classes of alteration were found to be involved. Across different cancer types, SV-mediated alterations (Figure S2) involved receptor tyrosine kinase (RTK) pathway-related genes (*KRAS*, *ERBB2*), p53/Rb-related genes (*CCND1*, *CCNE1*, *CDK4*, *CDKN2A*, *MDM2*), *TERT*, MYC family genes (*MYC*, *MYCN*), and mTOR pathway-related genes (*PTEN, STK11*). Across the entire POG570 cohort, assessment of genes within pathways (Figure 1D) demonstrated a high number of SV-mediated alterations (either by fusion or altered cis-regulation) involving p53 or Rb (32 tumors out of 570, or 5.6%), RTKs (32, 5.6%), PI3K/AKT/mTOR (20, 3.5%), MYC family genes (32, 5.6%), and TERT (28, 4.9%). Other pathways considered but that did not involve SVs included Wnt/beta-catenin, SWI/SNF, chromatin modifiers, and HIPPO.

**Figure 2.**
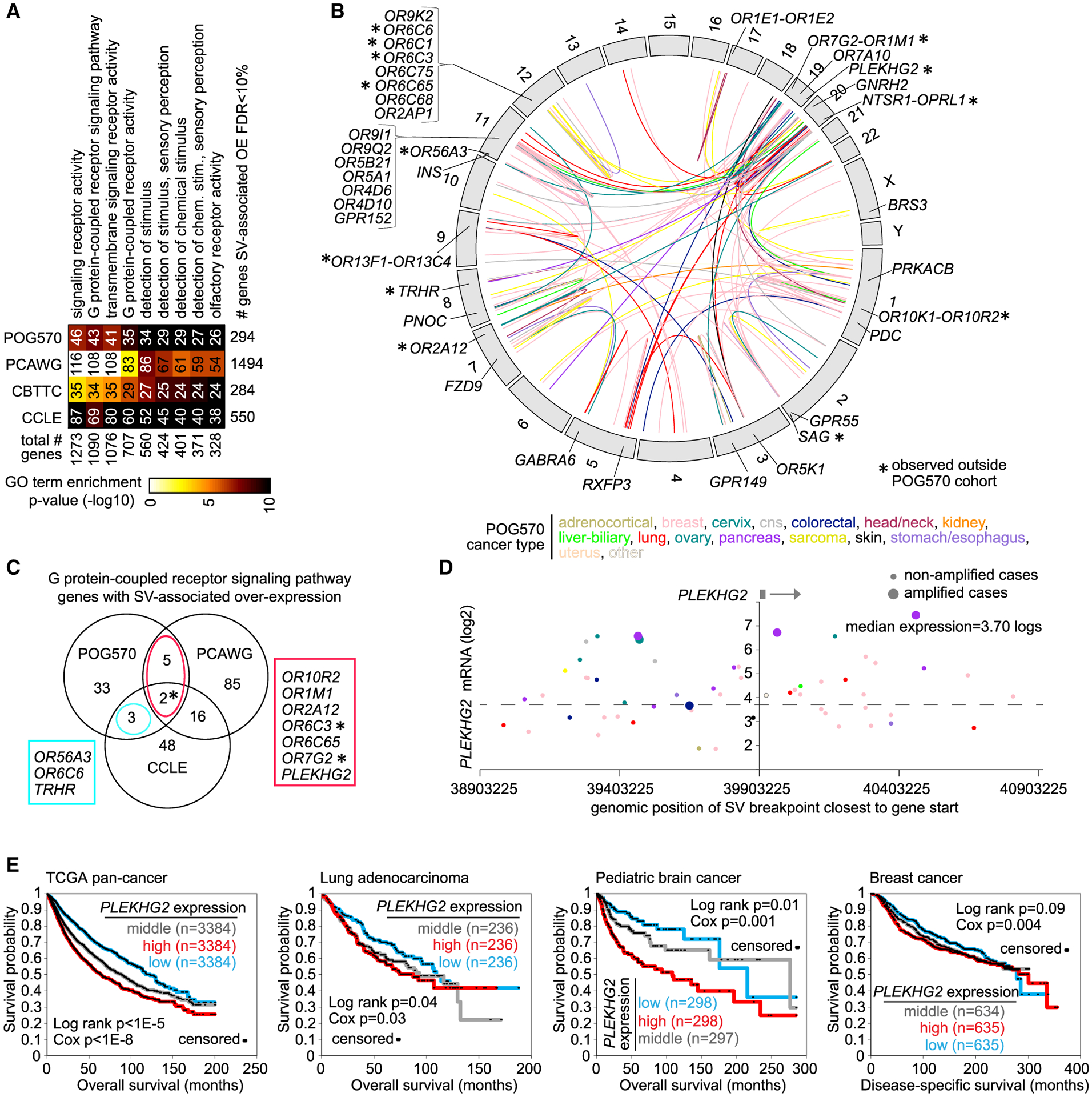
Somatic SV-mediated cis-regulatory alterations involve G-protein-coupled receptor signaling pathway genes (A) Selected significantly enriched Gene Ontology (GO) terms for genes with SV-expression associations (FDR < 10%, using a 1-Mb region, with corrections for tumor type and gene-level CNA), with gene set enrichment patterns (as indicated by degree of shading) evaluated separately for POG570, PCAWG, CBTTC, and CCLE cohorts. P values by one-sided Fisher’s exact test. (B) In the POG570 cohort, genomic rearrangements (represented in the circos plot) involving G-protein-coupled receptor signaling pathway genes. SV events are colored according to cancer type, as indicated. (C) Venn diagram of G-protein-coupled receptor signaling pathway genes with SV-associated overexpression (FDR < 10%, using a 1-Mb region) across multiple cohorts (POG570, PCAWG, CCLE). (D) In the POG570 cohort, gene expression levels of *PLEKHG2*, corresponding to SVs located in the genomic region 1 Mb downstream to 1 Mb upstream of the gene. Each point represents a single patient (closest SV breakpoint represented for each patient). Tumors with gene amplification are indicated. (E) Association of *PLEKHG2* mRNA expression with patient survival across multiple cancer types and four separate datasets: TCGA pan-cancer (n = 10,224), lung adenocarcinoma (n = 708), pediatric brain tumors (n = 893), and breast cancer (n = 1904). p values by log-rank test and by univariate Cox, as indicated. For TCGA pan-cancer and pediatric brain tumor datasets, p values are corrected for cancer type. See also Figure S3.

Of the 294 genes positively correlated with somatic SV breakpoints (1-Mb region window) across all cancers (FDR < 10%, correcting for cancer type and CNA), enriched Gene Ontology (GO) categories included signaling receptor activity (46 genes), G-protein-coupled receptor signaling pathway (43 genes), detection of chemical stimulus (29 genes), olfactory receptor activity (26 genes), and related GO terms (Figure 2A). Previous pan-cancer studies have associated olfactory receptor genes as a group with cancer cell differentiation status and prognosis (Kalra et al., 2020). Focusing here on the G-protein-coupled receptor signaling pathway genes (which highly overlap with the other categories above), we find SV-associated deregulation of these genes to involve many different cancer types in the POG570 cohort and most chromosomes (Figure 2B). Of the 570 POG570 tumors, 193 (34%) involved overexpression of one of the above G-protein-coupled receptor signaling pathway genes, coupled with an SV breakpoint (Figure S3). G-protein-coupled receptor genes were also enriched in the SV-associated genes from other cohorts (Figures 2A and 2C), but with some genes in this category being specific to each cohort. SV-altered G-protein-coupled receptor signaling pathway genes of interest included *PLEKHG2* (pleckstrin homology and RhoGEF domain containing G2; Figure 2D). Previously, mutation hotspots in the POG570 cohort were observed to involve the promoter of related gene *PLEKHS1* (Pleasance et al., 2020). Higher *PLEKHG2* mRNA expression was consistently associated with worse patient outcome across four large transcriptomic datasets (Figure 2E), representing a diverse range of cancer types and mostly primary tumors such as TCGA pan-cancer (Chen et al., 2018), lung adenocarcinoma (Chen et al., 2017b), pediatric brain tumors (Zhang et al., 2021b), and breast cancer (Pereira et al., 2016). In pancreatic cancer, *PLEKHG2* has a purported role as a 19q13 amplicon driver (Shain et al., 2013).

### SVs and associated genes involving patient survival

**Figure 3.**
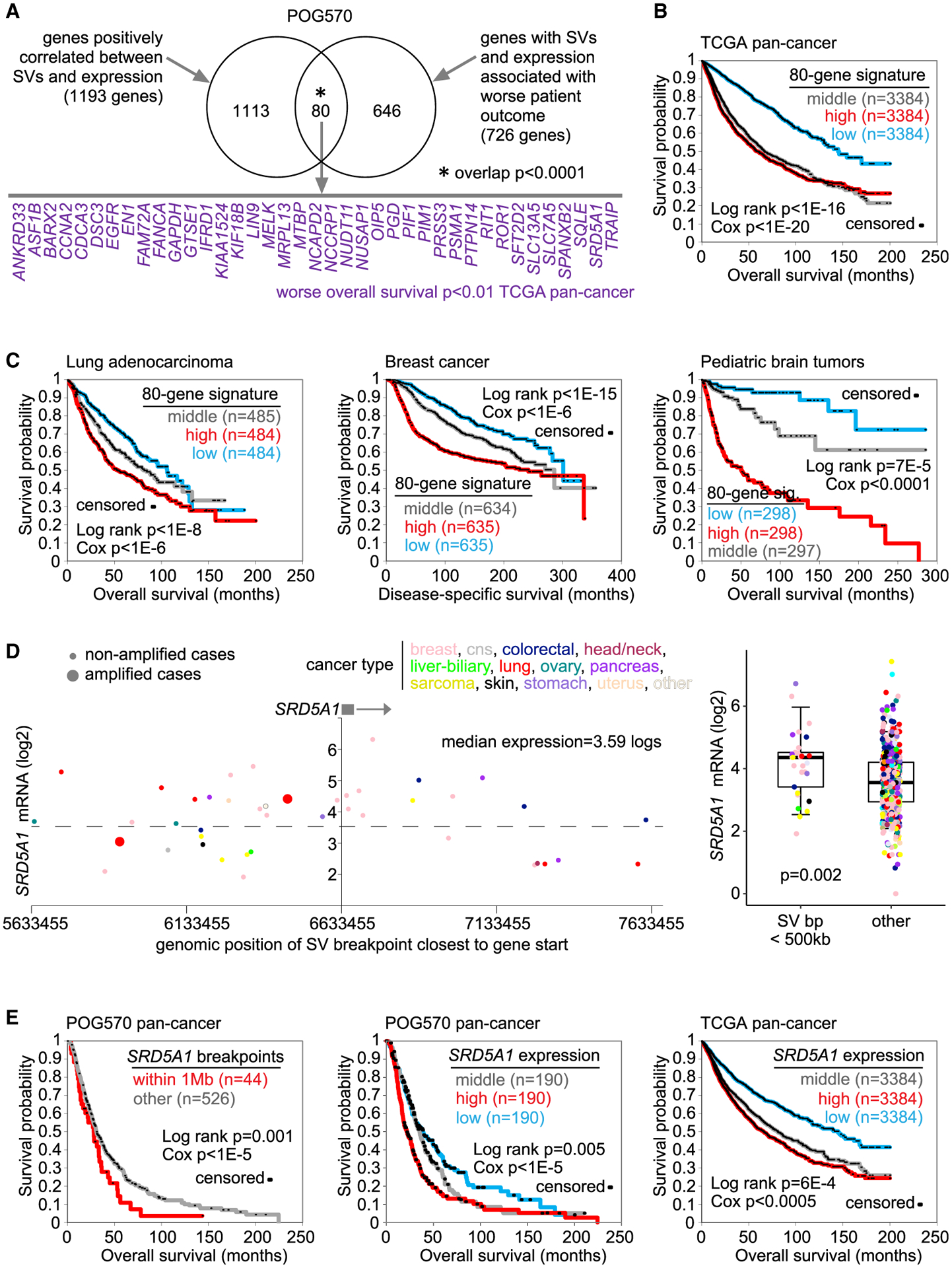
Somatic SV-mediated cis-regulatory alterations in the POG570 cohort involve patient survival (A) Of 1193 genes in the POG570 cohort with positive SV-expression associations (p < 0.05, using 1-Mb region, with corrections for tumor type and gene-level CNA), 80 had both nearby SV breakpoints and expression associated with worse patient outcome in the same cohort (p < 0.05 for each variable, stratified Cox correcting for cancer type; SV breakpoints also correcting for gene-level CNA). p value for significance of overlap by one-sided Fisher’s exact test. Genes with worse overall survival (p < 0.01, stratified Cox correcting for cancer type) in the TCGA pan-cancer dataset are listed by name. (B) Association of the 80-gene expression signature from (A) with patient survival in the TCGA pan-cancer dataset (n = 10,224). p values by log-rank test and by univariate Cox, as indicated, corrected for cancer type. (C) Association of the 80-gene expression signature from (A) with patient survival across multiple cancer types and three separate datasets: lung adenocarcinoma (n = 1453), pediatric brain tumors (n = 893), and breast cancer (n = 1904). p values by log-rank test and by univariate Cox, as indicated. For the pediatric brain tumor dataset, p values are corrected for histologic type. (D) In the POG570 cohort, gene expression levels of *SRD5A1*, corresponding to SVs located in the genomic region 1 Mb downstream to 1 Mb upstream of the gene (left). Each point represents a single patient (closest SV breakpoint represented for each patient). Tumors with gene amplification are indicated. The boxplot (right, representing 5%, 25%, 50%, 75%, and 95%) shows expression of *SRD5A1* by tumors with an SV breakpoint within 500 kb of gene start versus other tumors. p value by t test. (E) Association of *SRD5A1* SV breakpoint and expression patterns with worse patient outcomes in the POG570 cohort, and association of *SRD5A1* expression with worse patient outcomes in the TCGA pan-cancer cohort. p values by log-rank test and by univariate Cox, as indicated, both tests correcting for cancer type. See also Figure S4 and Table S4.

A significant fraction of the genes with SV-expression associations in the POG570 cohort also correlated with patient survival (Figures 3A and S4A). Across the POG570 cohort, 3394 and 3386 genes were associated with worse overall survival by somatic SV breakpoint pattern and expression, respectively (p < 0.05, stratified Cox analysis; Table S4; see STAR Methods), with 726 genes intersecting between both results sets. Taking a set of 1193 genes with positive SV-expression correlations (p < 0.05, correcting for cancer type and CNA, using more relaxed criteria from those of Figure 1A), 80 of these intersected with the 726 poor prognosis genes, a significant overlap (p < 0.0001, one-sided Fisher’s exact test). Among the 80 genes were just three with a well-established cancer association by COSMIC: *EGFR*, *FANCA*, and *PIM1*. Interestingly, we observed no overlap between genes with negative SV-expression correlation and genes associated with better overall survival.

The above 80-gene signature of SV-associated overexpression and worse patient survival, as defined using the POG570 cohort, was associated with worse patient outcomes across transcriptomic datasets representing a diverse range of cancer types and mostly primary tumors. Of the 80 genes, 39 individually associated with worse patient outcomes in the TCGA pan-cancer RNA-seq dataset of > 10,000 cancers and 32 cancer types (p < 0.01, stratified Cox by cancer type; Figure 3A), whereas just one gene was associated with better patient outcomes. We scored the TCGA tumors based on the entire 80-gene signature (taking the average of the normalized expression values in each tumor profile). In TCGA, the signature was associated with worse overall survival in this cohort of predominantly primary cancers (Figure 3B). Similarly, the 80-gene signature was associated with worse prognosis across additional datasets for lung adenocarcinoma, pediatric brain tumors, and breast cancer (Figure 3C). As one example of a gene in the 80-gene signature perhaps understudied in the context of cancer, *SRD5A1* (steroid 5 alpha-reductase 1 gene), involved in progesterone metabolism (Sinreih et al., 2015), showed an association of nearby SV breakpoints with increased expression (Figure 3D). *SRD5A1* also showed associations with worse patient survival in terms of both breakpoint patterns and expression for POG570 and worse survival associations for multiple external pan-cancer datasets, including TCGA (Figures 3E and S4B).

### SVs associated with TAD disruption, enhancer hijacking, and translocated retrotransposons

**Figure 4.**
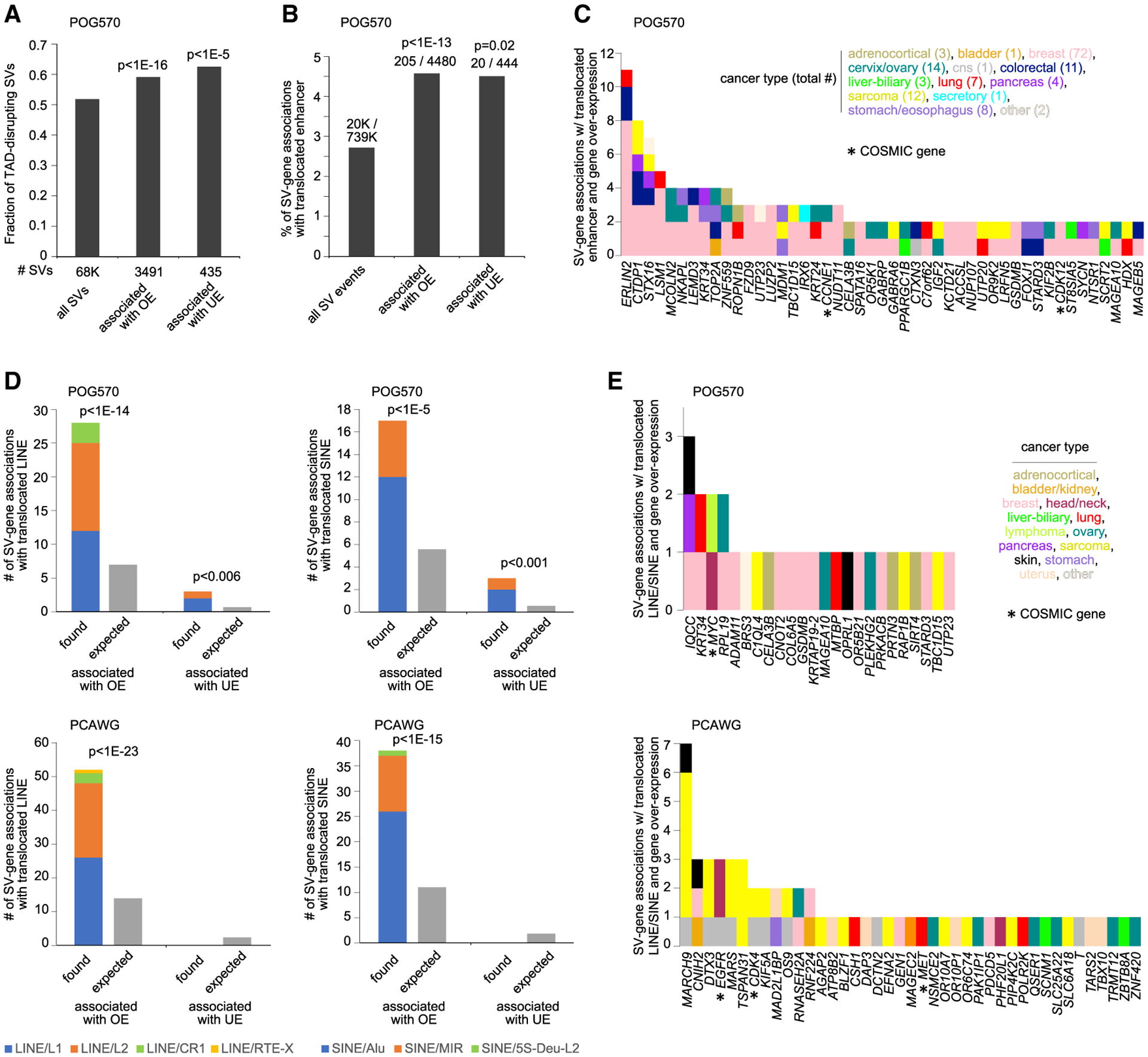
Somatic SVs associated with TAD disruption, enhancer hijacking, and translocated retrotransposons (A) Compared to all somatic SVs, fractions of SVs involving both TAD disruption and SV-associated altered gene expression, in the POG570 cohort. (B) For the POG570 cohort, percentages of SV breakpoint associations involving the translocation of an enhancer within 1 Mb of the SV breakpoint in proximity to the gene (and closer than any enhancer for the unaltered gene), as tabulated for all SV breakpoint associations as well as for the subsets involving altered gene expression. (C) By gene and by cancer type, the number of SV breakpoint associations from (B) involving the translocation of an enhancer and involving at least two patients per gene. Results involve 47 genes and 139 tumors. (D) For the POG570 cohort (top) and PCAWG cohort (bottom), numbers of SV breakpoint associations involving the translocation of a LINE or SINE retrotransposon within 20 kb of the SV breakpoint in proximity to the gene (and closer than any LINE or SINE for the unaltered gene, respectively), as tabulated for all SV breakpoint associations as well as for the subsets involving altered gene expression. Expected number of events based on probability. (E) For the POG570 cohort (top) and the PCAWG cohort (bottom), by gene and by cancer type, the number of SV breakpoint associations from (D) involving the translocation of LINE or SINE retrotransposons. For (A) to (E), SV-associated overexpression or underexpression is defined as an FDR < 10% for the gene using a 1-Mb region window, with corrections for cancer type and CNA, and expression > 0.4 SD or < −4 SD, respectively, from median for the case harboring the breakpoint. For (A), (B), and (D), enrichment p values by chi-square test. OE, overexpression; UE, underexpression. For (C) and (E), COSMIC genes are indicated. See also Figure S5 and Table S5.

Many possible mechanisms could conceivably be at work in SV-mediated gene deregulation patterns, including, but not limited to, disruption of TADs and enhancer hijacking. We examined the set of somatic SV events associated with overexpression in the POG570 cohort (FDR < 10%, using a 1-Mb region window, with corrections for cancer type and CNA, and expression > 0.4 SD from median for the case harboring the breakpoint). We found significant enrichment for TAD-disrupting SVs (SVs for which the breakpoints span two different TADs) (Figure 4A, p < 1E-16, chi-square test). Similarly, SV breakpoints involving gene overexpression were enriched (p < 1E-13, chi-square test) for putative enhancer hijacking events, with the rearrangement bringing an enhancer within 1 Mb of the gene (Figure 4B; Table S5), involving 113 overexpressed genes and 205 tumors, including *CCNE1* and *CDK12* (Figure 4C). The above findings for the POG570 cohort were consistent with similar findings for the PCAWG, TCGA, CBTTC, and CCLE cohorts (Zhang et al., 2019, 2021a, 2021b).

Another finding, not previously reported in our SV-expression integrative studies, involved significant numbers of retrotransposons—both long interspersed elements (LINEs) and short interspersed elements (SINEs)—being translocated near genes in tumors with SV-associated overexpression. LINEs and SINEs can regulate gene transcription by altering chromatin structure and functioning as enhancers or promoters (Elbarbary et al., 2016; Jang et al., 2019). In the POG570 cohort, SV breakpoints involving gene overexpression were enriched for events involving translocated LINEs or SINEs (p < 1E-14 and p < 1E-5, respectively, chi-square test), with the rearrangement bringing a LINE or SINE within 20 kb of the gene (Figure 4D, a shorter distance being considered here due to the shorter range of promoter effects). The phenomenon observed likely represents a type of promoter hijacking, with the rearrangement bringing the LINE or SINE from a different location in proximity to the gene. In the POG570 cohort, the above involved 24 overexpressed genes and 29 tumors, including *MYC* (Figures 4E and S5A; Table S5). Similar findings involving translocated LINEs or SINEs were observed here in each of the PCAWG, CBTTC, and CCLE cohorts (Figures 4D, 4E, S5B, and S5C), considerably strengthening this global association involving a fraction of SV-mediated deregulation events. The above phenomenon would be entirely distinct from that of somatic retrotransposition in cancer, the latter involving retrotransposon elements copying and inserting themselves at new genomic sites (Rodriguez-Martin et al., 2020).

### Associations involving chromothripsis and overall SV burden

**Figure 5.**
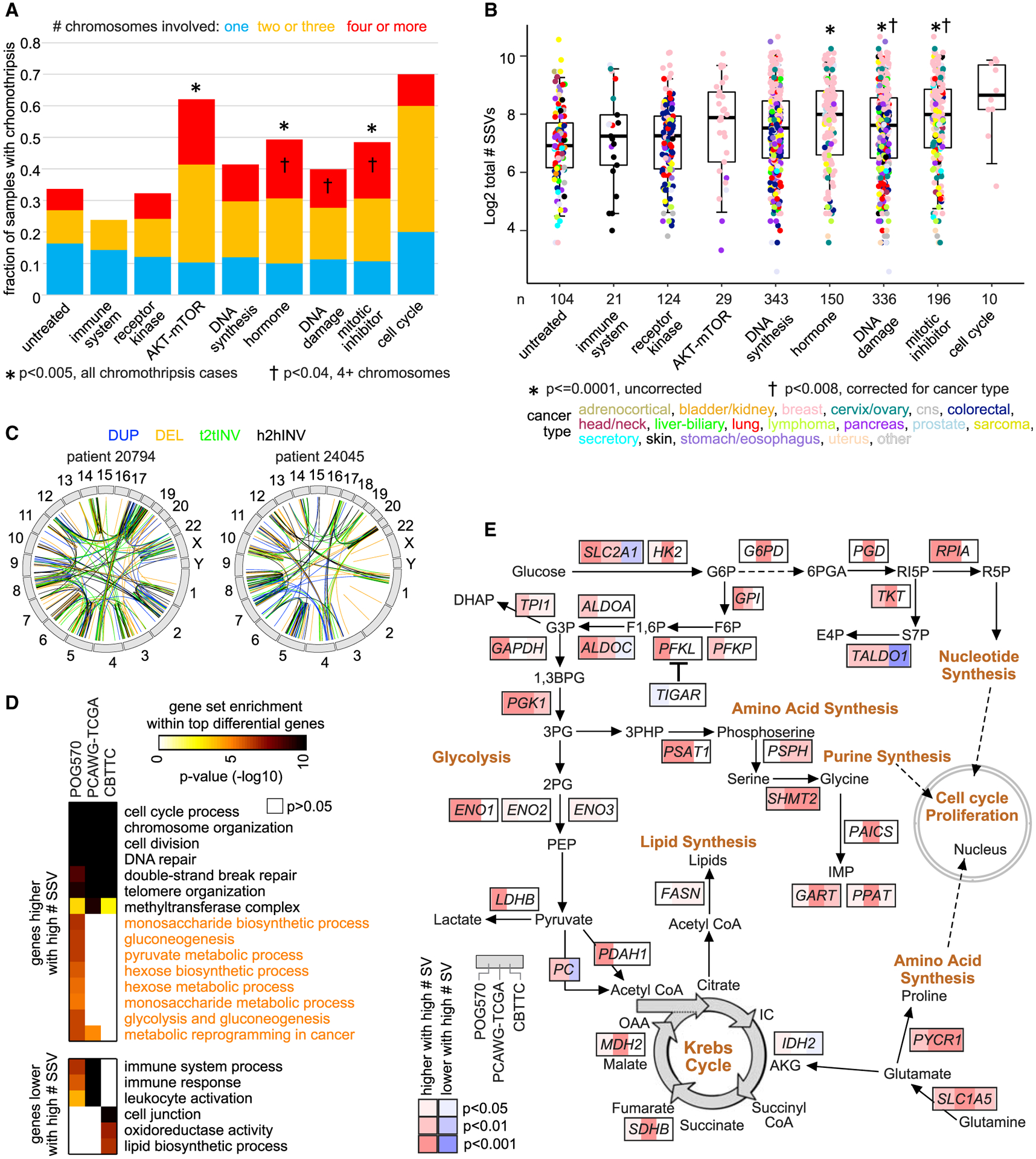
Associations of overall SV burden and chromothripsis with patient therapy (A) Fraction of patients with chromothripsis events, according to patient treatment group (by targeted pathway). Enrichment p values (comparing each treatment group with the rest of the tumors) by one-sided Fisher’s exact test. (B) Total number of SVs detected in the tumor, according to patient treatment group (by targeted pathway). p values (comparing each treatment group with the rest of the tumors) by t test or regression model incorporating cancer type, as indicated. The boxplot represents 5%, 25%, 50%, 75%, and 95%. (C) Circos plots representing two examples of tumors from taxane-treated patients with chromothripsis events involving 11 different chromosomes. (D) Selected significantly enriched GO terms and wikiPathways (Slenter et al., 2018) for genes correlated (FDR < 10%, with corrections for tumor type and gene-level CNA) with the total number of SVs, with enrichment patterns (as indicated by degree of shading) evaluated separately for POG570, PCAWG-TCGA (Zhang et al., 2019), and CBTTC datasets. p values by one-sided Fisher’s exact test. (E) Pathway diagram representing core metabolic pathways, with corresponding correlations with the overall structural variation burden within POG570, PCAWG-TCGA, and CBTTC datasets (red, significantly higher with an increasing number of SVs, correcting for tumor type and CNA). See also Figure S6 and Table S6.

We found broad associations between high overall somatic SV burden and prior treatment of patients with either DNA damage inducers (i.e., DNA alkylating agents) or mitotic inhibitors (taxanes in particular; Figures 5A, 5B, and S6). Chromothripsis, a phenomenon characterized by massive genomic rearrangements affecting one or more chromosomes, was empirically assessed in the POG570 cancers using the ShatterSeek method (Cortés-Ciriano et al., 2020), with 212 of 570 tumors exhibiting chromothripsis in at least one chromosome (Table S1). We considered nine patient treatment groups defined by targeted pathway (including the set of 104 untreated patients), with tumors biopsied after therapy. Tumors from patients treated with DNA damage inducers (DNA alkylating agents specifically) or mitotic inhibitors (86% of which involved taxanes) had a higher representation of chromothripsis events and higher overall numbers of SVs. While tumor mutational burden (SNVs and in-dels) was previously associated with exposure to genotoxic therapy (Pleasance et al., 2020), treatments targeting DNA synthesis in particular (including DNA synthesis inhibitors, anthracyclines, and topoisomerase I/II inhibitors) were not associated here as a group with higher SV burden or more chromothripsis events relative to the other therapy groups, although anthracyclines and DNA alkylating agents were (Figure S6). Chromothripsis tended to be greater in extent in the therapy groups involving either DNA damage/DNA alkylating agents or mitotic inhibitors/taxanes as well as hormone-related pathways. Fourteen POG570 tumors each had chromothripsis in seven or more chromosomes, with 10 tumors involving taxanes or anthracyclines (Figure 5C).

Following a similar line of investigation as that of our previous studies (Zhang et al., 2019, 2021b), we examined gene expression features in POG570 that correlated with the total number of somatic SV breakpoints detected per tumor, independently of where the breakpoints fall in proximity to genes (Table S6). At an FDR < 10% (linear regression model, correcting for tumor type and gene-level CNA), 1784 genes showed an increase and 807 genes showed a decrease, with overall SV burden. We observed common patterns of differential genes and gene categories associated with SV burden across the POG570, PCAWG, and CBTTC cohorts (Figure 5D). For the increased genes, all three cohorts showed high enrichment for genes related to cell cycle process, chromosome organization, cell division, DNA repair including double-strand break repair, telomere organization, and methyltransferase complex. For the decreased genes, both POG570 and PCAWG cohorts showed enrichment for immune response-related genes. Interestingly, in contrast to the PCAWG and CBTTC cohorts, genes increased with SV burden in the POG570 cohort involved core metabolic pathways, including gluconeogenesis, pyruvate metabolism, hexose biosynthesis and metabolism, monosaccharide metabolism, and glycolysis and gluconeogenesis. In surveying curated genes and pathways related to metabolic reprogramming in cancer, most of these genes were increased in the POG570 cohort in association with high SV burden, with many of these genes also showing a corresponding association in PCAWG or CBTTC cohorts or both (Figure 5E).

### SVs and associated genes involving patient therapy

**Figure 6.**
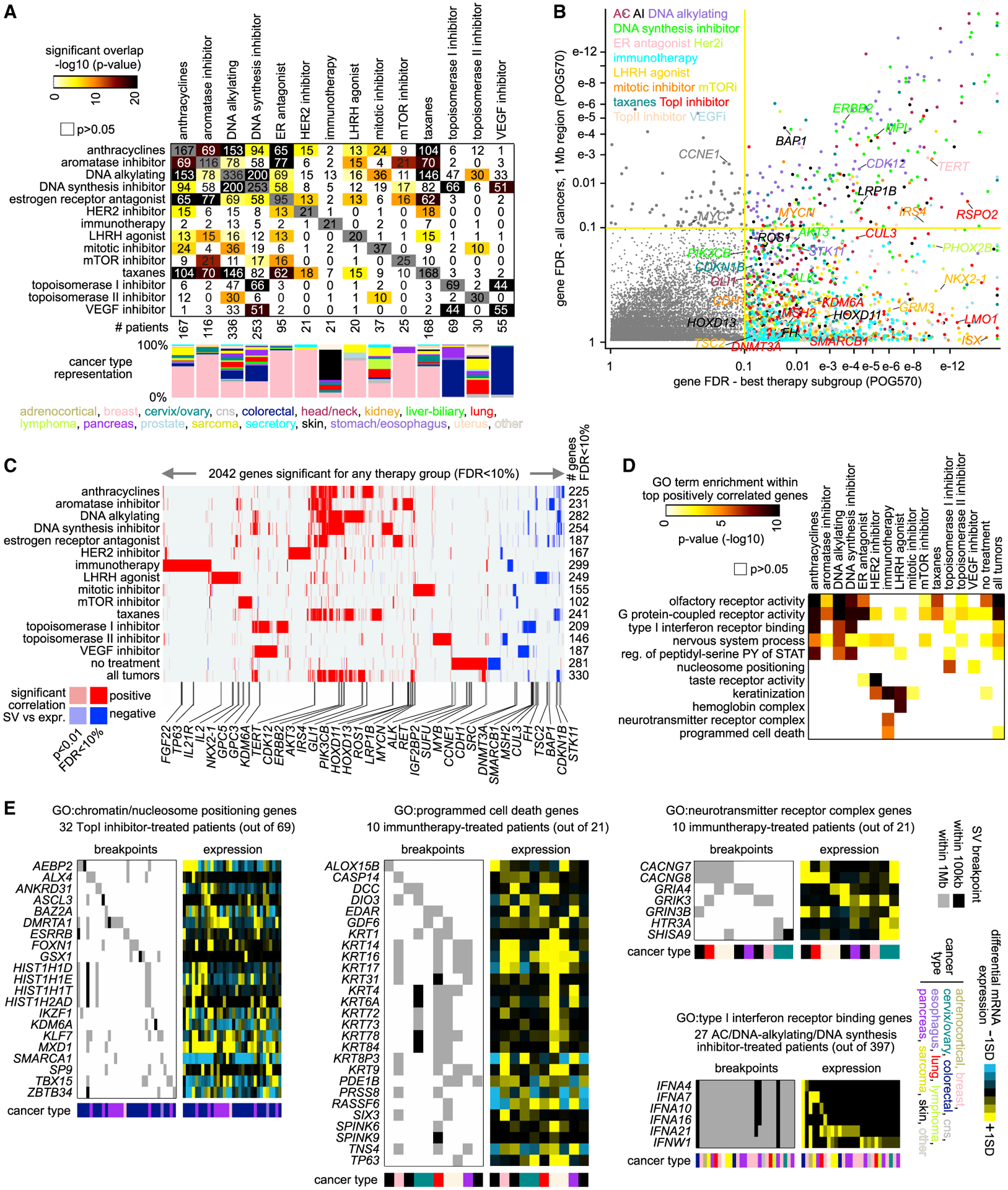
Somatic SV-expression associations within therapy subgroups (A) Major therapy subgroups (by therapeutic agent) present in the POG570 cohort, representing both the degree of overlap between therapy subgroups and breakdown according to cancer type. (B) The x axis indicates the SV-expression association FDR in the most significant of 14 therapy subgroups analyzed separately, and the y axis indicates the FDR when the entire POG570 cohort is analyzed together. Genes in the upper left quadrant reached significance only in the full cohort analysis. Genes in the lower right quadrant reached significance only in one or more therapy-specific analyses. The color of the data points represents the most significant tumor type. AC, anthracycline; AI, aromatase inhibitor; ER, estrogen receptor. (C) Heatmap of differential t-statistics by therapy subgroup, evaluating gene expression alterations with nearby SV breakpoints (red, positive correlation with breakpoint; white, not significant), for 2042 genes significant for one or more individual therapy subgroups (FDR < 10%, see STAR Methods). Selected COSMIC genes are listed by name. (D) According to the therapy subgroup, selected significantly enriched GO terms for genes positively correlated (FDR < 10%) with nearby SV breakpoints. p values by one-sided Fisher’s exact test. The set of GO terms represented were significant (FDR < 10%) within the top genes for at least one therapy subgroup. (E) Patterns of SV versus expression for selected GO term gene sets and therapy subgroups from (D). Tumors represented for each gene set and therapy subgroup are those for which an SV breakpoint was within 1 Mb of the gene and for which the gene had expression levels greater than the sample median. For (B) through (E), significant genes with SV-expression associations are defined by a 1-Mb region window, correcting for tumor type and CNA. See also Figure S7 and Table S7.

In addition to analyzing all 570 tumors in the POG570 cohort together, we carried out analyses to define gene-level SV-expression associations within POG570 subgroups defined according to patient therapy (Table S7). Most patients (n = 466, 82%) in the POG570 cohort received systemic therapy before biopsy and genomic analysis, involving 110 different drugs. We defined 14 different patient therapy subgroups involving sizeable numbers of patients for analysis by SVExpress (Figure 6A). Many patients received more than one therapy. For example, many breast cancer patients received both estrogen receptor (ER) antagonist and aromatase inhibitor therapy, and many patients received more than one genotoxic therapy (e.g., anthracyclines, DNA alkylating agent, DNA synthesis inhibitor). For each therapy subgroup, hundreds of genes showed significant SV-expression associations (FDR < 10%; STAR Methods), and many genes significant in the analysis of therapy-specific subgroups did not reach significance when analyzing the entire POG570 cohort (Figure 6B). These therapy-specific associations would be analogous to results from pan-cancer studies surveying significantly mutated genes (Lawrence et al., 2014) or significant SV associations (Zhang et al., 2019), whereby some genes may be significant within a given cancer subset but not for the entire pan-cancer cohort. For many therapy subgroups, the respective significant gene sets appeared entirely distinct from those of the other subgroups and the entire cohort (Figures 6C and S7). For some subgroups for which there was high patient overlap between them, including the examples noted above, there were high overlaps in significant gene patterns as might be expected.

Functional gene categories of interest, as defined by GO, were significantly enriched (FDR < 10%) within the top genes for at least one therapy subgroup (Figure 6D). Many of these gene categories were enriched specifically within the respective results for one or two therapy subgroups and less enriched within the entire POG570 cohort. Significantly enriched gene categories (p < 0.0005, one-sided Fisher’s exact test) included nucleosome positioning and chromatin genes for topoisomerase I inhibitor-treated patients, programmed cell death genes and neurotransmitter receptor complex genes for immunotherapy-treated patients, and type I interferon receptor binding genes in patients treated with anthracyclines, DNA alkylating agents, or DNA synthesis inhibitors (Figure 6E). Interestingly, a connection between topoisomerase I inhibitor treatment and increased chromatin accessibility has been established elsewhere (Baranello et al., 2010). Within a given tumor subgroup, genes with significant SV-associated alterations may involve breakpoints in just one or two samples, combined with very high expression. When tabulating the altered expression events across individual genes, however, the above functional gene groups would involve a substantial fraction of patients within a given therapy subgroup (e.g., roughly half of immunotherapy-treated patients and of topoisomerase I inhibitor-treated patients).

**Figure 7.**
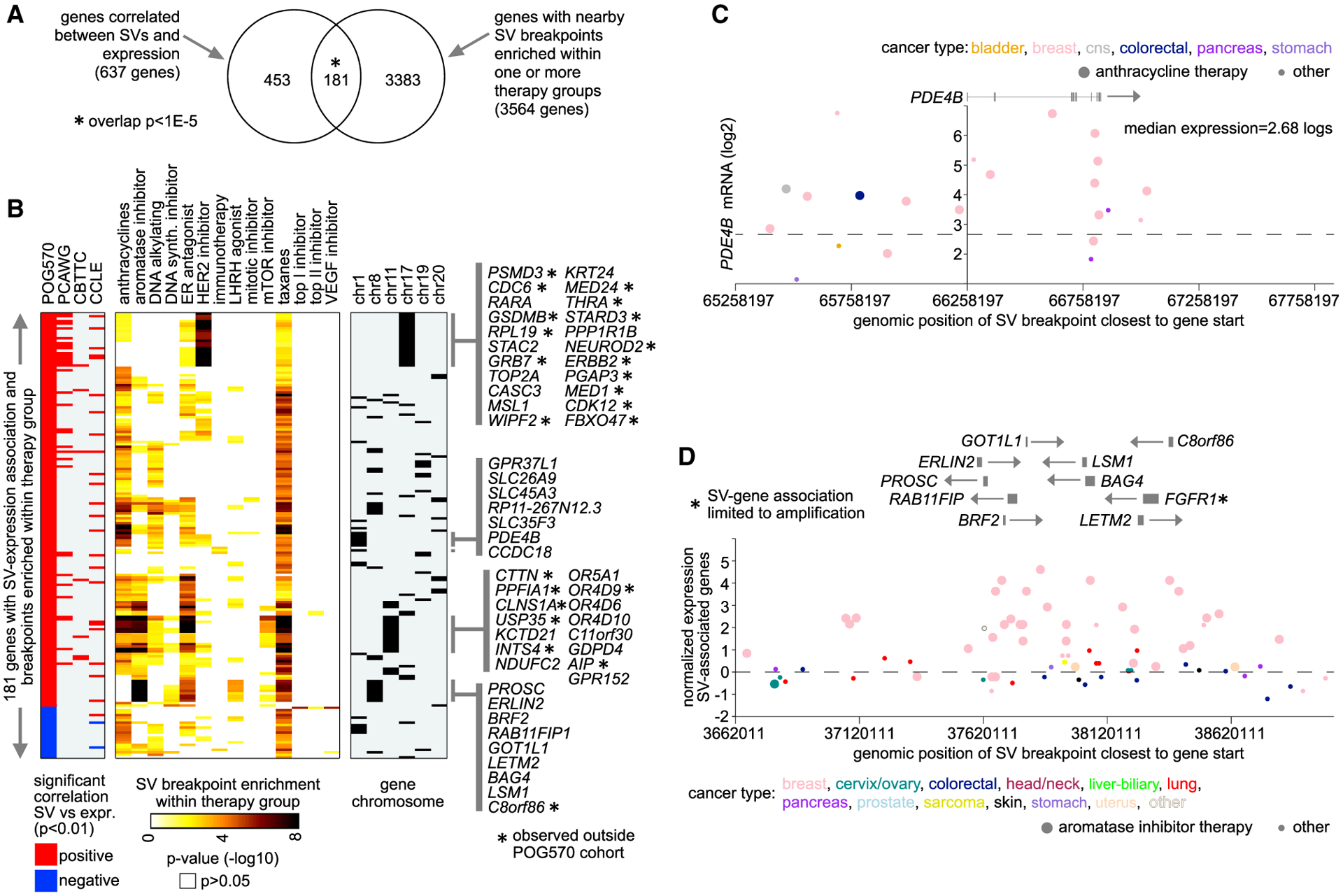
Somatic SV-mediated cis-regulatory alterations with breakpoints enriched within therapy subgroups (A) Of 637 genes in the POG570 cohort with SV-expression associations (p < 0.01, using a 1-Mb region, with corrections for tumor type and gene-level CNA), 181 had SV breakpoints (within 1 Mb of the gene) enriched within one or more therapy subgroups (p < 0.001). p value for significance of overlap by one-sided Fisher’s exact test. (B) The 181 genes from (A), with corresponding gene-level SV-expression associations for POG570, PCAWG, CBTTC, and CCLE cohorts (red, significant positive correlation; blue, significant negative correlation; p < 0.01, correcting for cancer type and CNA), and with corresponding statistical enrichment of breakpoint events (occurring within 1 Mb of the gene) by therapy subgroup (from Figure 6A). Specific clusters of genes associated with a specific therapy subgroup may all be located within the same chromosomal region, as indicated. (C) In the POG570 cohort, gene expression levels of *PDE4B* corresponding to SVs located in the genomic region 1 Mb downstream to 1 Mb upstream of the gene. Each point represents a single patient (closest SV breakpoint represented for each patient). Tumors from patients treated with anthracyclines are indicated. (D) In the POG570 cohort, averaged normalized expression levels of genes in the 8p11.23 cytoband region from (B), corresponding to SVs located in the genomic region 1 Mb downstream to 1 Mb upstream of the indicated genes. Each point represents a single breakpoint (closest SV breakpoint to a significant gene represented for a given patient). In all, 215 breakpoints from 119 patients are represented. Multiple breakpoints from the same patient will have the same level of expression. Tumors from patients treated with aromatase inhibitors are indicated. See also Table S7.

As another line of investigation, we re-examined the set of genes with SV-expression associations across the entire POG570 cohort for genes with somatic SV breakpoint patterns also associated with patient therapy. We considered 637 genes in the POG570 cohort with significant SV-expression associations (p < 0.01, using the 1-Mb region, with corrections for tumor type and gene-level CNA). Of these 637 genes, 181 had nearby SV breakpoints (within 1 Mb) enriched (p < 0.001, one-sided Fisher’s exact test) within one or more therapy groups, representing a highly significant fraction (p < 1E-5, one-sided Fisher’s exact test; Figure 7A; Table S7). For most of the 181 genes, the SV-expression association was found only for the POG570 cohort but not for the other cohorts examined (Figure 7B). One notable exception involved numerous genes in the *ERBB2* amplicon region within 17q. *ERBB2* and related genes were significant (after correcting for amplification) for both POG570 and PCAWG cohorts, as well as showing SV breakpoint enrichment within HER2 inhibitor-treated tumors in the POG570 cohort. *ERBB2* (HER2) amplifications represent a clinical biomarker for HER2-inhibitor therapy, and many of the breakpoint patterns as observed in POG570 advanced cancers would conceivably have involved the primary cancer. Most of the 181 genes had enrichment for associated breakpoints within taxane-treated patients, likely reflecting the increased tumor SV burden within this subgroup (mitotic inhibitors; Figures 5A, 5B, and S6).

Somatic SV breakpoint enrichment patterns of interest included genes on chromosome 1, including *PDE4B*, associated with anthracycline treatment (Figures 7B and 7C), and 8p11.23 genes associated with aromatase or ER inhibitor therapy (Figures 7B and 7D). *PDE4B*, part of the PDE4 subfamily of phosphodiesterases, for which there is an emerging role in malignancy, has associated inhibitors approved for treating inflammatory diseases (Hsien Lai et al., 2020). POG570 tumors with SV breakpoints near *PDE4B* involved 22 patients, 16 of which had breast cancer and 15 of which were treated with anthracycline (Figure 7C). As the chromosome 1 genes would not have been clinical biomarkers for anthracyclines, SV events involving *PDE4B* and its nearby genes (none of which had SV-expression associations observed in the PCAWG primary tumors) could represent selection in resistant tumors. The 8p11.23 genes associated with aromatase or ER inhibitor therapy involved multiple genes for which SV-expression associations, independent of gene-level CNA, were found (Figure 7D). The 8p11.2 amplicon is associated with early relapse in ER-positive breast cancer (Bilal et al., 2012) as well as with the more aggressive luminal B molecular subtype of ER-positive breast cancer (Voutsadakis, 2020). The enrichment for 8p11.23-associated breakpoints within patients treated with therapies targeting the ER pathway would signify an over-representation of luminal B over luminal A ER-positive tumors in the setting of advanced disease. Some nine genes in the 8p11.23 region involve SV-associated overexpression, independent of CNA, involving 119 patients, 47 of them treated with aromatase inhibitor therapy (Figure 7D). Interestingly, 8p11.2 gene *FGFR1*, implicated in hormonal resistance in ER+ breast cancer, had a significant SV-expression association but one that was dependent on amplification. Other genes in this region—including *BAG4*, *BRF2*, *ERLIN2*, *GOT1L1*, *LETM2*, *LSM1*, *PROSC*, and *RAB11FIP1*—all had SV-expression associations independent of CNA, some of which likely also play a role in advanced disease (Streicher et al., 2007; Wang et al., 2012).

## DISCUSSION

In the setting of advanced cancers with complex treatment histories, we observed widespread associations between altered gene expression and the presence of nearby somatic SV breakpoints, representative of rearrangement-mediated cis-regulatory alterations. Not all deregulated genes observed may play an important role in the disease. However, the present study could identify important SV-expression associations by focusing on the following: well-established oncogenes and tumor suppressor genes, enriched gene classes, patient survival associations, associations with prebiopsy therapy, and corresponding SV-expression associations in external cohorts. Across multiple patient cohorts, G-protein-coupled receptor genes, including olfactory receptor genes, were found to be consistently enriched among SV-associated overexpressed genes. Ectopically expressed olfactory receptors have been linked with multiple clinically relevant physiological processes, including epithelial malignancies (Kalra et al., 2020), and more investigation into their potential role in cancer is arguably warranted.

In addition to TAD disruption and enhancer hijacking, translocation of retrotransposons, as uncovered in this study, represents a likely mechanism involving some of the SV-mediated altered cis-regulation patterns observed. Beyond their ability to duplicate themselves and jump around their host genomes, retrotransposons such as LINEs or SINEs are now increasingly understood as regulating gene expression. SINEs and LINEs can promote as well as inhibit the transcription of nearby genes, for example, by acting as an enhancer, alternate promoter, or transcription start site (Elbarbary et al., 2016). Regulatory elements within transposable elements can, for example, be epigenetically reactivated in cancer (Jang et al., 2019). As the POG570-related patterns involving enhancer hijacking or translocated retrotransposons demonstrate, our analytical approach represents a platform for discovering additional mechanisms of SV-mediated altered cis-regulation, in terms of enrichment of genetic elements involving the subset of SV-expression associations.

According to therapy, we could identify SV-expression associations within patient subgroups, with a number of these associations not being significant across the entire POG570 cohort. For example, we found that tumors from patients treated with topoisomerase I inhibitors have a significant number of SV-expression associations involving chromatin-related genes. Elsewhere, topoisomerase I inhibition has been found to trigger transcriptional stress, involving antisense transcription and increased chromatin accessibility (Baranello et al., 2010). SV breakpoint enrichment patterns by therapy subgroup, such as SV-associated *PDE4B* overexpression within anthracyclin-treated tumors as observed here, could point to pathways of resistance, thereby suggesting combined therapeutic strategies.

### Limitations of the study

While we could identify hundreds of genes with robust SV-expression associations across 570 advanced cancers, there would be a great advantage in profiling much larger numbers of tumors from treated patients with combined WGS and RNA-seq. For a given gene, the therapy subgroup-specific associations may involve just one or two samples within a smaller tumor subset. In this case, it would be difficult to identify the most relevant SV-expression associations, although the observed enriched gene categories may point to intriguing hypotheses. Future studies can leverage larger numbers of samples to identify more robust associations, with therapy-specific associations less likely to be confounded by other variables.

## STAR★METHODS

### RESOURCE AVAILABILITY

#### Lead contact

Further information and requests for resources and reagents should be directed to and will be fulfilled by the lead contact, Chad J. Creighton (creighto@bcm.edu).

#### Materials availability

This study did not generate new unique reagents.

#### Data and code availability

This paper analyzes existing, publicly available data. Details on accessing the datasets are listed in the Key Resources Table. Genomic and transcriptomic sequence datasets for the POG570 cohort, including metadata with library construction and sequencing approaches have been deposited at the European Genome–phenome Archive (EGA, https://www.ebi.ac.uk/ega/) as part of the study EGAS00001001159 with accession numbers as listed in (Pleasance et al., 2020). Data on mutations, copy changes and expression from tumor samples in the POG program organized by OncoTree classification (http://oncotree.mskcc.org) are also accessible from https://www.personalizedoncogenomics.org/cbioportal/. The complete small mutation catalog and gene expression TPMs are available for download from https://bcgsc.ca/downloads/POG570/.

CBTTC molecular data are available through the public project on the Kids First Data Resource Portal and Cavatica (https://cbtn.org/) and through the PedCBioPortal (https://pedcbioportal.org/). PCAWG data are available at the ICGC Data Portal (https://dcc.icgc.org/pcawg). The CCLE datasets are available at https://portals.broadinstitute.org/ccle/data.

This paper does not report original code. No custom computer code was used for data collection, which was performed using open-source software. Additional processing involved in-house scripts that are available upon request. All analyses used previously published software or methods. SVExpress is freely available at https://github.com/chadcreighton/SVExpress.

Any additional information required to reanalyze the data reported in this paper is available from the lead contact upon request.

### EXPERIMENTAL MODEL AND SUBJECT DETAILS

Regarding human subjects, cancer molecular profiling data were generated through informed consent as part of previously published studies and analyzed in accordance with each original study’s data use guidelines and restrictions.

### METHOD DETAILS

#### Patient cohorts

Results are based upon data generated as part of the Personalized OncoGenomics (POG) Program at BC Cancer (Pleasance et al., 2020). Combined WGS analysis (at 80x coverage, minimum of 45x for the 570 tumors, with average read alignment of 92%) and RNA-seq analysis (at 40x coverage, minimum of 26x) was carried out for 570 tumor samples from 570 patients as previously described (Pleasance et al., 2020). Tumor molecular profiling data were generated through informed consent as part of POG efforts and analyzed here per POG’s data use guidelines and restrictions. The POG570 cohort is composed of advanced and metastatic tumors from patients treated in a tertiary cancer clinic and represents 23 major cancer types: adrenocortical (n = 4), bladder (n = 1), breast (n = 144), cervix (n = 4), cns (n = 19), colorectal (n = 87), eosophagus (n = 10), head/neck (n = 7), kidney (n = 5), liver-biliary (n = 16), lung (n = 67), lymphoma (n = 11), ovary (n = 28), pancreas (n = 42), prostate (n = 3), sarcoma (n = 47), secretory (n = 12), skin (n = 22), stomach (n = 11), uterus (n = 11), and miscellaneous (n = 11). Most biopsies were taken from metastatic sites (n = 438, 77%), whereas others represented local recurrences or refractory disease.

Most patients in the POG570 cohort (n = 466, 82%) received systemic therapy before biopsy. Therapy categories involving larger numbers of patients were considered in subgroup analyses, including subgroups based on ‘Class I’ therapeutic agent designations (Pleasance et al., 2020) (anthracyclines, n = 167 patients; aromatase inhibitor, n = 116; DNA alkylating, n = 336; DNA synthesis inhibitor, n = 253; ER antagonist, n = 95; HER2 inhibitor, n = 21; LHRH agonist, n = 20; mitotic inhibitor, n = 37; mTOR inhibitor, n = 25; taxanes, n = 168; topoisomerase I inhibitor, n = 69; topoisomerase II inhibitor, n = 30; VEGF inhibitor, n = 55) and subgroups based on targeted pathway (AKT-mTOR, n = 29; cell cycle, n = 10; DNA damage, n = 336; DNA synthesis, n = 343; hormone, n = 150; immune system, n = 21; mitotic inhibitor, n = 196; receptor kinase, n = 124).

The results here are also based in part upon data generated by TCGA Research Network, the International Cancer Genomics Consortium (ICGC), the CBTTC, and the CCLE project. Previously, we carried out combined WGS and RNA-seq analysis for 2334 TCGA-ICGC cancer cases in total(Zhang et al., 2019) (1232 of which were part of the PCAWG consortium efforts), a patient cohort referred to in the present study as the “PCAWG-TCGA” cohort. The PCAWG cohort involves the 1220 patient tumors with combined WGS and RNA-seq data that comprised the final freeze datasets of the PCAWG consortium (ICGC-TCGA Pan-Cancer Analysis of Whole Genomes Network, 2020; Zhang et al., 2020). The PCAWG dataset was analyzed here using SVExpress to define gene-level SV-expression associations within a 1Mb region surrounding each gene. Most cancers represented in PCAWG were from primary cancers. Of the 1220 PCAWG patients with combined WGS and RNA-seq data, just 53 (4.3%, most of these being melanoma) involved metastatic or recurrent tumors. The CBTTC datasets consisted of combined WGS and RNA-seq on 854 pediatric brain tumors from 759 patients, with SV-expression associations generated previously (Zhang et al., 2021b). The CCLE datasets, consisting of 327 cancer cell lines with combined WGS and expression data, were analyzed previously for SV-expression associations (Zhang et al., 2021a).

#### Molecular profiling datasets

For the POG570 datasets, sequence reads from normal and tumor whole genome libraries were aligned to the human reference genome (hg19). Tumor genome sequences were compared to those from the patient’s constitutive (normal) DNA to identify somatic alterations. Somatic single nucleotide variants (SNVs) and small (< 20 bp) insertions and deletions (indels) were identified as previously described (Pleasance et al., 2020). SVs at the DNA level, based on WGS data, were identified using assembly-based tools ABySS and Trans ABySS and alignment-based tools Manta v1.0.0 (Chen et al., 2016) and Delly v0.7.3 (Rausch et al., 2012). Putative SV calls were merged into a consensus caller MAVIS (v2.1.1) (Reisle et al., 2019), where they were clustered, computationally validated, and annotated against constitutional DNA to provide somatic and germline structural variant calls. SV calls were additionally filtered to identify those called by more than one tool, and for which a contig could be assembled that aligned across a candidate genomic breakpoint. Germline SV calls were removed from the dataset. Somatic SV calls were further filtered to exclude events with identical genomic breakpoints in multiple samples, removing potentially confounding germline variants and technical artifacts. Previous efforts had identified 1,815 potential gene fusion events across the POG570 cohort (Pleasance et al., 2020), with support by both WGS SV data and detection of chimeric transcripts by RNA-seq. RNA-seq reads were aligned and gene-level expression was quantified as transcripts per million (TPM) as previously described (Pleasance et al., 2020), with gene annotations based on Ensembl v.85. WGS-based gene-level copy values were generated as integers representing the predicted copy number state using CNAseq (Jones et al., 2010).

#### Integrative analyses between SVs and expression

Using SVExpress (Zhang et al., 2021a), we defined genes with altered expression associated with nearby somatic SV breakpoints across the POG570 cohort. No germline SVs were used in any analyses. Relative to each gene, genomic region windows considered included the within-gene regions and within 100kb upstream or 100kb downstream of the gene. For the above regions, SVExpress constructed a gene-to-sample matrix with entries as 1, if a breakpoint occurs in the specified region for the given gene in the given sample, and 0 if otherwise. We also used SVExpress to examine a 1Mb region surrounding each gene, using the “relative distance metric” option (Zhang et al., 2019), whereby breakpoints that occur close to the gene will have more numeric weight in identifying SV-expression associations, while breakpoints further away but within 1Mb can have some influence. Gene-level SV-expression association analyses using the POG570 datasets included 16720 unique named genes. Using the geneXsample SV breakpoint matrix, SVExpress assessed the correlation between expression of the gene and the presence of an SV breakpoint using a linear regression model (with log-transformed expression values), incorporating sample cancer type (i.e., “histological_type”) and gene-level CNA. For the analyses involving within-gene, 100kb upstream, and 100kb downstream gene regions, we only considered genes with at least three tumors associated with an SV within the given region.

By SVExpress, a gene shows significant SV-expression associations if the expression and SV breakpoint patterns line up non-randomly with respect to each other across all samples analyzed, after correction for covariates. By design (Zhang et al., 2019), our analytical approach does not assume the specific mechanism of altered expression (as many diverse scenarios would be plausible, some of which, including TAD disruption and enhancer hijacking, were explored in this study). This aspect of SVExpress would include treating SV breakpoints representing different classes (tandem duplications, insertions, deletions, inversions, and translocations) and insert sizes the same in the integration with gene expression. In principle, somatic SVs of any class or size may lead to altered cis-regulation, depending on the specifics involving any nearby rearrangements and the gene regulatory landscape, and significant SV-gene associations by SVExpress involve all SV classes and sizes (Zhang et al., 2019, 2021b). SVExpress does not rely on the presence of SV breakpoint clusters or hotspot patterns for a gene to achieve significance; if multiple breakpoints occur near the gene, the breakpoint closest to the gene start is used in the breakpoint matrix. Tumor purity, tumor ploidy, and total number of SV breakpoints have been found to not represent significant confounders in identifying SV-expression associations (Zhang et al., 2020).

In addition to analyzing all 570 tumors in the POG570 cohort together, we carried out analyses within subgroups of the POG570 cohort, defined according to cancer type or patient therapy. Subgroups considered were those with sufficient numbers of tumors to provide adequate power to detect SV-expression associations, based on our previous studies (Zhang et al., 2019, 2021b). For the subgroup analyses, category subgroups with fewer than 19 or 20 tumors (for cancer type-specific and treatment-specific subgroup analyses, respectively) were not considered, as lack of power due to the sparse nature of SV breakpoints would be more of an issue with the smaller subgroups. For the therapy-based subgroup analyses, the subgroups were defined based on ‘Class I’ therapy designations, with the addition of an immunotherapy subgroup representing five different agents against PD-1, PD-L1, CTLA4, or OX40. The distance metric method (Zhang et al., 2019) with 1Mb gene region window was used to assess SV-expression associations within POG570 subgroups (including CNA and cancer type as covariates). In addition, for the therapy-specific subgroup analyses, we filtered statistically significant genes for those with at least one sample in the subgroup having high or low expression (> 0.4DS or < −0.4SD, respectively, from the sample median) combined with breakpoint within 1Mb of the gene. For some patient subgroups, genes with significant SV-associated alterations may involve breakpoints in just one or two samples, combined with very high or very low expression changes relative to the other tumors in the subgroup.

#### Pathway-level somatic alteration categories

For the pathway-centric view of somatic alterations (Figure 1D), key pathways and genes previously annotated across multiple cancer types based on domain knowledge (Cancer Genome Atlas Research Network, 2014; Chen et al., 2017b, 2018; Zhang et al., 2018) were included: Receptor Tyrosine Kinase (RTK) pathway (*BRAF*, *EGFR*, *ERBB2*, *ERBB3*, *ERBB4*, *FGFR1*, *FGFR2*, *FGFR3*, *FGFR4*, *HRAS*, *KIT*, *KRAS*, *MET*, *NF1*, *NRAS*), HIPPO pathway (*NF2*, *SAV1*, *WWC1*), chromatin modification (*CREBBP*, *EHMT1*, *EHMT2*, *EP300*, *EZH1*, *EZH2*, *KAT2A*, *KAT2B*, *KDM1A*, *KDM1B*, *KDM4A*, *KDM4B*, *KDM5A*, *KDM5B*, *KDM5C*, *KDM6A*, *KDM6B*, *KMT2A*, *KMT2B*, *KMT2C*, *KMT2D*, *KMT2E*, *NSD1*, *SETD2*, *SMYD4*, *SRCAP*), SWI/SNF complex (*ACTB*, *ACTL6A*, *ACTL6B*, *ARID1A*, *ARID1B*, *ARID2*, *BCL11A*, *BCL11B*, *BCL6*, *BCL6B*, *BRD7*, *BRD9*, *DPF1*, *DPF2*, *DPF3*, *PBRM1*, *PHF10*, *SMARCA2*, *SMARCA4*, *SMARCB1*, *SMARCC1*, *SMARCC2*, *SMARCD1*, *SMARCD2*, *SMARCD3*, *SMARCE1*), mTOR pathway (*AKT1*, *AKT2*, *AKT3*, *MTOR*, *PIK3CA*, *PIK3R1*, *PTEN*, *RHEB*, *STK11*, *TSC1*, *TSC2*, *IDH1*, *IDH2*, *VHL*), MYC family (*MYC*, *MYCN, MYB*), *TERT*, Wnt/beta-catenin (*APC*, *AXIN1*, *CTNNB1*, *FGF19*, *NCOR1*), and p53/Rb-related (*ATM*, *CCND1*, *CCNE1*, *CDK4*, *CDKN1A*, *CDKN2A*, *E2F2*, *E2F3*, *FBXW7*, *MDM2*, *RB1*, *TP53*). For known oncogenes (e.g., *AKT1*, *MTOR*, *PIK3CA*, *RHEB*, *BRAF*, *EGFR*, *ERBB2*, *ERBB3*, *HRAS*, *KRAS*, *NRAS*), if an SNV occurred in “hotspot” residues as reported by Chang et al. (2016), the SNV was considered in the analysis. We considered all inactivating SNVs (nonstop/nonsense) and indels in putative tumor suppressor genes (e.g., *TP53*) in the analyses. We also considered *TERT* activating promoter mutations (Huang et al., 2013). At both the gene and pathway levels, we tabulated somatic alterations in the following order: SNV or indel, gene fusion, deep deletion (estimated zero gene copies), high-level amplification (estimated five or more copies), and somatic SV (for oncogenes, breakpoint falling with 1Mb of gene and associated with expression > 0.4SD from median for the given tumor; for tumor suppressors, breakpoint falling within the gene body and expression < −0.4SD). SVs were considered only for those genes significant (FDR < 10%) by SV-expression analyses for either the gene body region, 100kb upstream region, 100 downstream region, or the 1MB region (incorporating cancer type and CNA).

#### Survival analyses

We identified molecular correlates of patient survival in the POG570 cohort. For associating nearby SV breakpoints with patient outcome, we utilized the [gene X tumor] relative distance breakpoint matrix, as generated by SVExpress. For each gene, we used a stratified Cox (correcting for cancer type and gene-level CNA) to associate patient overall survival with the log2-transformed relative distance to the nearest breakpoint for that gene. We also associated expression of the gene with overall survival using stratified Cox (corrected for cancer type). Genes significant for both the relative breakpoint analysis and the expression analysis were compared with the set of genes with SV-expression associations as determined by SVExpress. When overlapping different results sets, a more relaxed p value cutoff was used to limit false negatives, as the degree of gene set overlap itself was found significant, as well as yielding significant results in multiple external datasets. The numbers of significant genes associated with worse survival by either expression or breakpoint pattern far exceeded chance expected (see Results), and in taking the 726 genes that overlap between both expression and breakpoint survival results sets (Figure 3A), multiple testing becomes even less of a concern. In contrast to the POG570 cohort, the PCAWG cohort had a median follow-up time (~2.4 years) that would be considered short for the purposes of deriving robust survival correlates (at least for primary cancer patients).

Genes and gene sets of interest, as identified through analysis of the POG570 cohort, were examined in public cancer transcriptomic datasets, for associations between expression and patient outcome. For analysis of lung adenocarcinoma patient survival, we examined a compendium dataset of 11 published mRNA expression profiling datasets for human lung adenocarcinomas (Chen et al., 2016; Monsivais et al., 2021). For analysis of breast cancer patient survival, we used the Pereira et al. expression dataset (Pereira et al., 2016) (as downloaded from CBioPortal). For analysis of pediatric brain tumor patient survival, we used RNA-seq data from CBTTC (Monsivais et al., 2021; Zhang et al., 2021b). The TCGA pan-cancer RNA-seq dataset, representing 32 major cancer types and 10224 tumors, was assembled as previously described (Chen et al., 2018). Given a gene signature (e.g., the 80-gene signature of Figure 3A), we scored patient profiles in the external expression dataset by taking the average of the normalized expression values (standard deviations from the median across samples) for the entire set of genes. We assessed the association of the expression of individual genes or a gene signature score with patient outcome using univariate Cox and log-rank (dividing the cases according to low, high, or intermediate signature scoring). In addition, for analyses utilizing the TCGA pan-cancer or CBTTC datasets, stratified Cox models or stratified log-rank were used to evaluate survival association when correcting for tumor type. For analyses involving the lung adenocarcinoma compendium or TCGA pan-cancer datasets, patient survival was capped at 200 months. For CBTTC dataset, only one tumor per patient was included in the survival analyses, and patient survival was capped at 285 months.

#### Integration of TADs, enhancers, and retrotransposons

To identify breakpoints associated with TAD disruption, we used the SVExpress (Zhang et al., 2021a) ‘Generate_SV_to_TAD_Associations’ module with published TAD data from the IMR90 cell line (Dixon et al., 2012), and using the UCSC Genome Browser LiftOver tool to convert TAD coordinates from hg18 to hg19. We defined TAD-disrupting SVs as those SVs for which the two breakpoints did not fall within the same TAD.

To identify potential enhancer hijacking events involving gene-level SV-expression associations, we used the SVExpress ‘Generate_SV_to_Enhancer_Associations’ module with the enhancer annotations as provided by Kumar et al. (Kumar et al., 2020). For each SV breakpoint association within 1Mb upstream of a gene (as provided by the SVExpress ‘Generate_Gene_to_Sample_SV_Table’ module, each association involving unique breakpoint and gene pairing, with only the SV breakpoint closest to the start of each gene being considered for each tumor in the instance of multiple breakpoints being detected), SVExpress determined the potential for translocation of an enhancer near the gene that would be represented by the rearrangement (based on the orientation of the SV breakpoint mate). SVExpress considered only SVs with breakpoints on the distal side from the gene in this analysis.

To identify translocation of LINE and SINE retrotransponsons involving SVs associated with altered gene expression, we again used the SVExpress ‘Generate_SV_to_Enhancer_Associations’ module but using the annotations from RepeatMasker open v4.0.5 (hg19) and v4.0.5 (hg38, for CBTTC dataset) (Smit et al., 2013–2015). For each SV breakpoint association within 20kb upstream of a gene, SVExpress determined the potential for translocation of a retrotransposon near the gene that would be represented by the rearrangement (based on the orientation of the SV breakpoint mate). SVExpress considered only SVs with breakpoints on the distal side from the gene in this analysis. The phenomenon of translocated retrotransposons as studied here is distinct from that of somatic retrotransposition (Rodriguez-Martin et al., 2020).

#### Associations with genomic rearrangement burden

We used ShatterSeek (Cortés-Ciriano et al., 2020) to identify regions of chromothripsis across the POG570 tumor samples. Chromosomes with chromothripsis were defined using the high confidence criteria according to ref (Cortés-Ciriano et al., 2020), with either one of the following being met: 1) at least six interleaved intrachromosomal SVs, seven contiguous segments oscillating between two CN states, the fragment joins test, and either the chromosomal enrichment or the exponential distribution of breakpoints test; or (2) at least three interleaved intrachromosomal SVs and four or more interchromosomal SVs, seven contiguous segments oscillating between two CN states and the fragment joins test. We then carried visual confirmation of the ShatterSeek calls, by examining ShatterSeek plots of copy and SV patterns for each chromosome region. Differences in the numbers of samples with chromothripsis events, by therapy subgroup according to targeted pathway, were examined, as well as the numbers of samples with chromothripsis involving four or more chromosomes.

We assessed differential gene expression patterns associated with the overall burden of structural variation across the POG570 tumors, as we did in our previous studies of other cancer cohorts (Zhang et al., 2019, 2021b). SV calls made for each tumor profiled were tabulated. For each gene, we assessed the correlation between expression and the total number of SV events detected across the 570 tumors. We used linear regression models with both log-transformed expression values and log-transformed SV event numbers, correcting for gene-level CNA and cancer type.

### QUANTIFICATION AND STATISTICAL ANALYSIS

All p values were two-sided unless otherwise specified. Individual molecular features were evaluated for correlation with patient survival by univariate Cox analysis or log-rank test. In addition, for pan-cancer or pan-histology datasets (e.g., POG570, TCGA, CBTTC), stratified Cox models or stratified log-rank were used to evaluate survival association when correcting for tumor type. As part of SVExpress, linear modeling was performed using the lm function in R (version 4.0.3). For linear regression modeling, t test, and Cox analyses, appropriate data transformations (log2 transform) were used to make the data align better with model assumptions. One-sided Fisher’s exact tests or chi-square tests determined significance of overlap between two given feature lists. The method of Storey and Tibshirani (2003) estimated FDR for significant genes, using the following formula for each gene: [(nominal p value) × (total number of genes tested)/(total number of genes that were significant at the given p value)]. Visualization using heatmaps was performed using JavaTreeview (Saldanha, 2004) and matrix2png (version 1.2.1) (Pavlidis and Noble, 2003). Enrichment of GO annotation terms and wikiPathway (Slenter et al., 2018) gene sets within sets of differentially expressed genes was evaluated using SigTerms software (Creighton et al., 2008) and one-sided Fisher’s exact tests. Gene sets for each wikiPathway were downloaded in July 2019 (“20190710” version). The method of Storey and Tibshirani (2003) estimated FDR for enriched gene sets. Figures indicate exact value of n (number of tumors), and the statistical tests used are noted in the Figure legends and next to reported p values in the Results section. Boxplots represent 5%, 25%, 50%, 75%, and 95%.

## Supplementary Material

1

2

3

4

5

6

7

8

## Figures and Tables

**Table:** KEY RESOURCES TABLE

REAGENT or RESOURCE	SOURCE	IDENTIFIER
Deposited Data		
POG570 sequencing data	European Genome-phenome Archive (EGA)	EGAS00001001159
POG570 small mutation and expression data	Canada’s Michael Smith Genome Sciences Centre (GSC) at BC Cancer	https://bcgsc.ca/downloads/POG570/
PCAWG genomic data	ICGC Data Portal	https://dcc.icgc.org/pcawg
CBTTC genomic data	Kids First Data Resource Portal and Cavatica	https://cbtn.org/informatics-portals
CCLE datasets	Broad Institute	https://portals.broadinstitute.org/ccle/data
Software and algorithms		
SVExpress	GitHub	https://github.com/chadcreighton/SVExpress
ABySS (v1.3.4)	GitHub	https://github.com/bcgsc/abyss
TransABySS (v1.4.10)	GitHub	https://github.com/bcgsc/transabyss
Chimerascan (v0.4.5)	Google Code Archive	https://code.google.com/archive/p/chimerascan/
DeFuse (v0.6.2)	GitHub	https://github.com/amcpherson/defuse
Manta (v1.0.0)	GitHub	https://github.com/Illumina/manta
Delly (v0.7.3)	GitHub	https://github.com/dellytools/delly
MAVIS (V2.1.1)	GitHub	https://github.com/bcgsc/mavis

## Highlights

- Combined structural variation and RNA analysis of 570 recurrent or metastatic tumors
- Retrotransposons translocated near genes is one mechanism of altered cis-regulation
- Structural variation breakpoint patterns enriched within prebiopsy therapy subgroups
