# Supplementary material for: Rearrangement-mediated cis-regulatory alterations in advanced patient tumors reveal interactions with therapy
Source: Cell Rep. Author manuscript; Available in PMC 2021 Nov 30. (PMC8630779; doi:10.1016/j.celrep.2021.110023)
Supplement: 1 [file NIHMS1757901-supplement-1.pdf]

Cell Reports, Volume *37*

Supplemental information

Rearrangement-mediated cis-regulatory alterations in advanced patient tumors reveal interactions with therapy

Yiqun Zhang, Fengju Chen, Erin Pleasance, Laura Williamson, Cameron J. Grisdale, Emma Titmuss, Janessa Laskin, Steven J.M. Jones, Isidro Cortes-Ciriano, Marco A. Marra, and Chad J. Creighton

Supplementary Figures

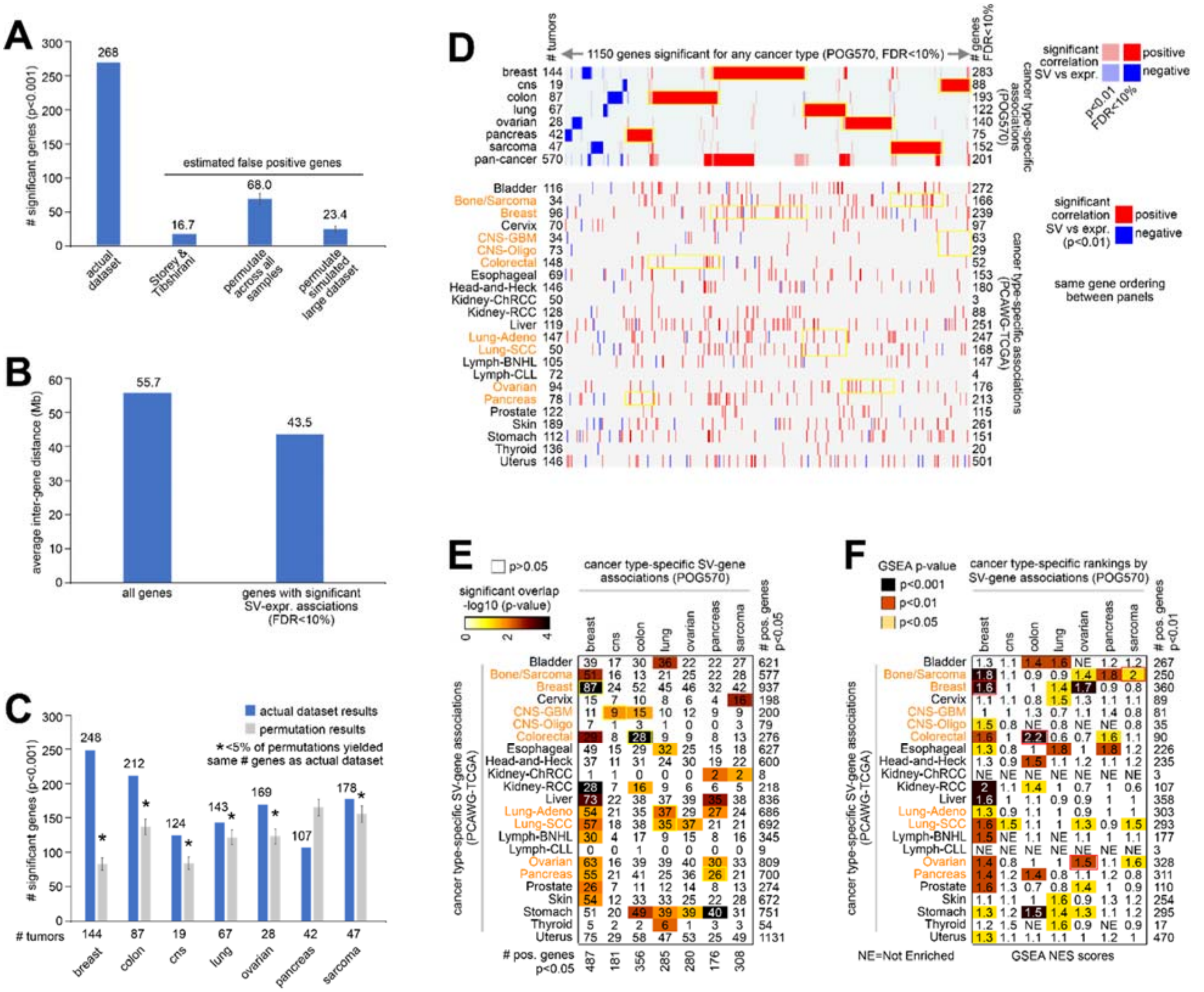

Figure S1. Additional information regarding genes with altered expression associated with nearby somatic SV breakpoints. Related to Figure 1. (A) Across the entire POG570 cohort, estimated false discovery rates (FDRs) involving the top significant genes with somatic SV-expression associations (p<0.001, distance metric method using 1Mb region, correcting for both cancer type and CNA). FDRs are estimated using the following methods: 1) Storey and Tibshirani method(Storey and Tibshirani, 2003), 2) permutation of SV profiles across all 570 samples with respect to the expression profiles (1000 permutations), 3) permutation of SV profiles with respect to the expression

profiles but using a large simulated dataset (100 permutations). For the large simulated dataset, 25 copies of the actual 570-tumor matrices were concatenated together to make one large dataset of 14250 samples, where the same relationships as represented in the 570-tumor dataset are present, but with the larger sample size allowing for more permutations that have little or no overlap with the actual dataset (being closer to a truly random distribution). Error bars represent standard deviations. As expected, the large simulated dataset shows fewer estimated false positive genes as compared to permutation results using the original 570-tumor dataset, where a larger sample size affords more power. The Storey and Tibshirani method was used to estimate FDR in all other aspects of the study. **(B)** The set of genes having significant SV-expression associations (FDR<10%, distance metric method using 1Mb region, correcting for both cancer type and CNA) tended to be closer in distance to each other. For both all genes, and the subset of significant genes, we assessed all inter-gene distances (using the gene start position) for each chromosome. **(C)** Within indicated cancer types of the POG570 cohort, numbers of statistically significant genes with SV-expression association ($p<0.001$ by distance metric method using 1Mb region, correcting for CNA). Permutation results based on permutation of somatic SV profiles across all samples of the given cancer type. Error bars represent standard deviations. **(D)** Heat map of association t-statistics by cancer type in POG570 cohort, evaluating gene expression alterations with nearby SV breakpoint within the given cancer type (red, positive correlation with breakpoint; white, not significant with $p>0.01$), for 1150 genes significant for one or more individual cancer types (FDR<10% using 1Mb region, correcting for CNA). By cancer type, we found 75 to 283 significant genes (FDR<10%, correcting for CNA). Notably, many genes significant in analyzing individual cancer types did not reach significance when analyzing the combined pan-cancer set, consistent with previous studies (Zhang et al., 2021; Zhang et al., 2019). For these POG570 genes, corresponding cancer type-specific SV-expression association t-statistics in the PCAWG-TCGA cohort (derived previously (Zhang et al., 2019)) are represented. **(E)** By one-sided Fisher's exact test, significance of overlap between the POG570 cancer type-specific associations and the PCAWG-TCGA cancer type-specific associations (defined using $p<0.05$ and 1Mb region, correcting for CNA). Significance of overlap between the two cohorts is observed here for the results sets involving breast, colon, lung, and pancreas cancer types. **(F)** By Gene Set Enrichment Analysis (GSEA), significance of enrichment of the PCAWG-TCGA cancer type-specific gene sets within the genes ranked according to SV-expression association in POG570 by each cancer type. NES, normalized enrichment score. Significance of enrichment between the two cohorts is observed here for the results sets involving breast, colon, ovarian, and sarcoma cancer types. In comparing the respective

gene-level associations between the POG570 and PCAWG-TCGA cohorts, we observed overall levels of agreement between the results sets within each respective cancer type. Differences between the two sets of results likely originate in part from both the different cancers represented in the respective datasets and the sparse nature of SV events. SV-expression associations would involve SV events impacting just a fraction of samples. When examining a smaller subset of tumors from the larger cohort, sparse events impacting a fraction of tumors in one cohort by chance may not be present in the other cohort.

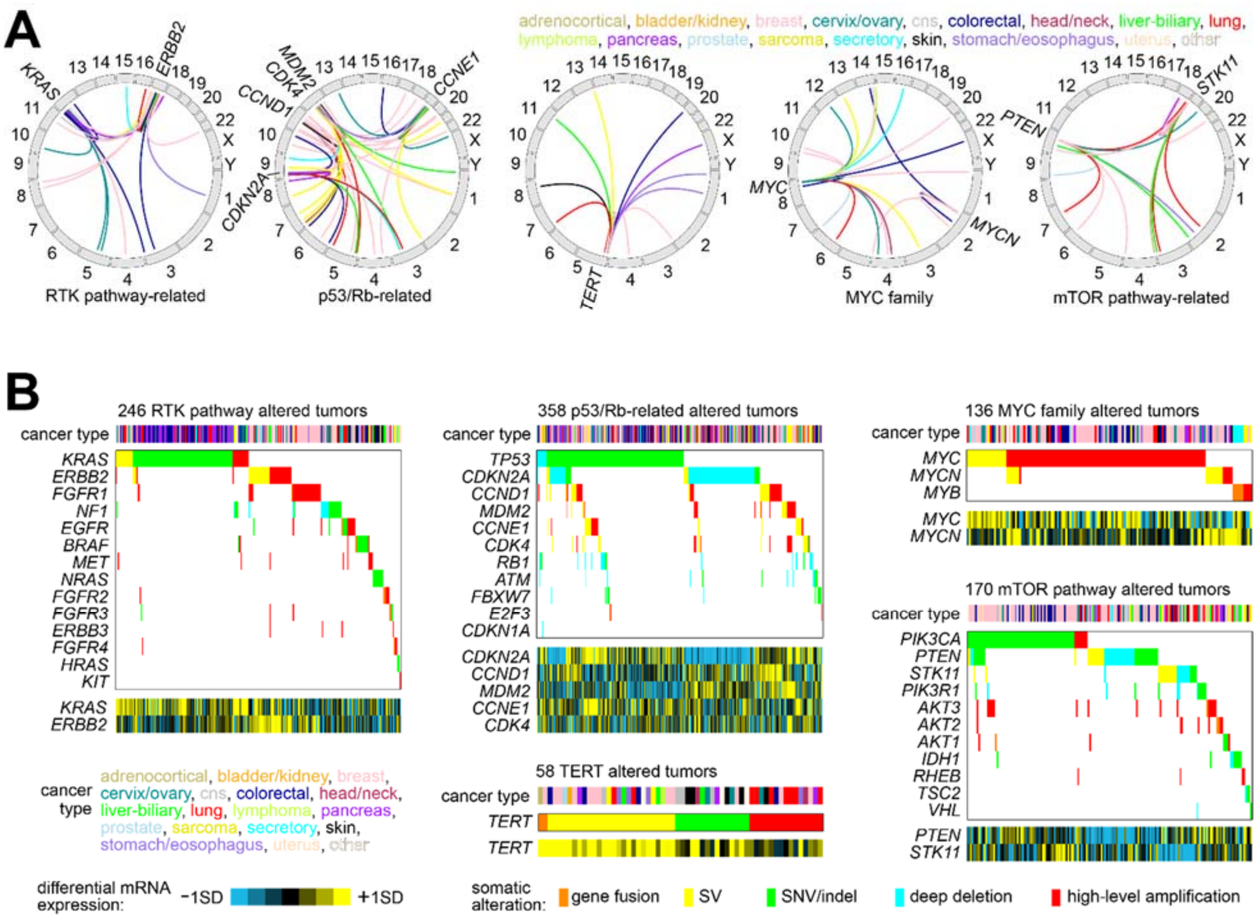

Figure S2. Somatic SVs in the POG570 cohort involving key oncogenic or tumor-suppressive pathways. Related to Figure 1. (A) Genomic rearrangements (represented in circos plots) involving Receptor Tyrosine Kinase (RTK) pathway-related genes (*KRAS*, *ERBB2*), p53/Rb-related genes (*CDK4*, *MDM2*, *CCND1*, *CCNE1*, *CDKN2A*), *TERT*, MYC family genes (*MYC*, *MYCN*), and mTOR pathway-related genes (*PTEN*, *STK11*). The above genes had globally significant SV-expression associations (Figure 1C). SV events represented had breakpoints occurring within 1Mb of the given gene, with altered expression for the sample (see below). SV events are colored according to cancer type, as indicated. **(B)** For the pathways from Figure 1d that also involve at least one SV event, somatic alteration events involving each gene included in the pathway are represented. For SV-impacted genes from part a, the corresponding differential mRNA expression patterns are shown. For part B and main Figure 1D, events are colored according to the type of somatic alteration: gene fusion, SV (for oncogenes, breakpoint falling with 1Mb of gene and associated with expression>0.4SD from median for the given tumor; for tumor suppressors, breakpoint falling within the gene body and expression<-0.4SD), SNV or indel (for oncogenes, SNV

within hotspot residue (Chang et al., 2016); for tumor suppressor genes, SNV within hotspot residue or inactivating mutation by indel/nonsense/nonstop), and deep deletion or high-level amplification (respectively approximating total copy loss and five or more copies). Other alteration classes not considered here, such as DNA methylation, could also conceivably impact these pathways (e.g., through p16 or VHL silencing (Chen et al., 2018)).

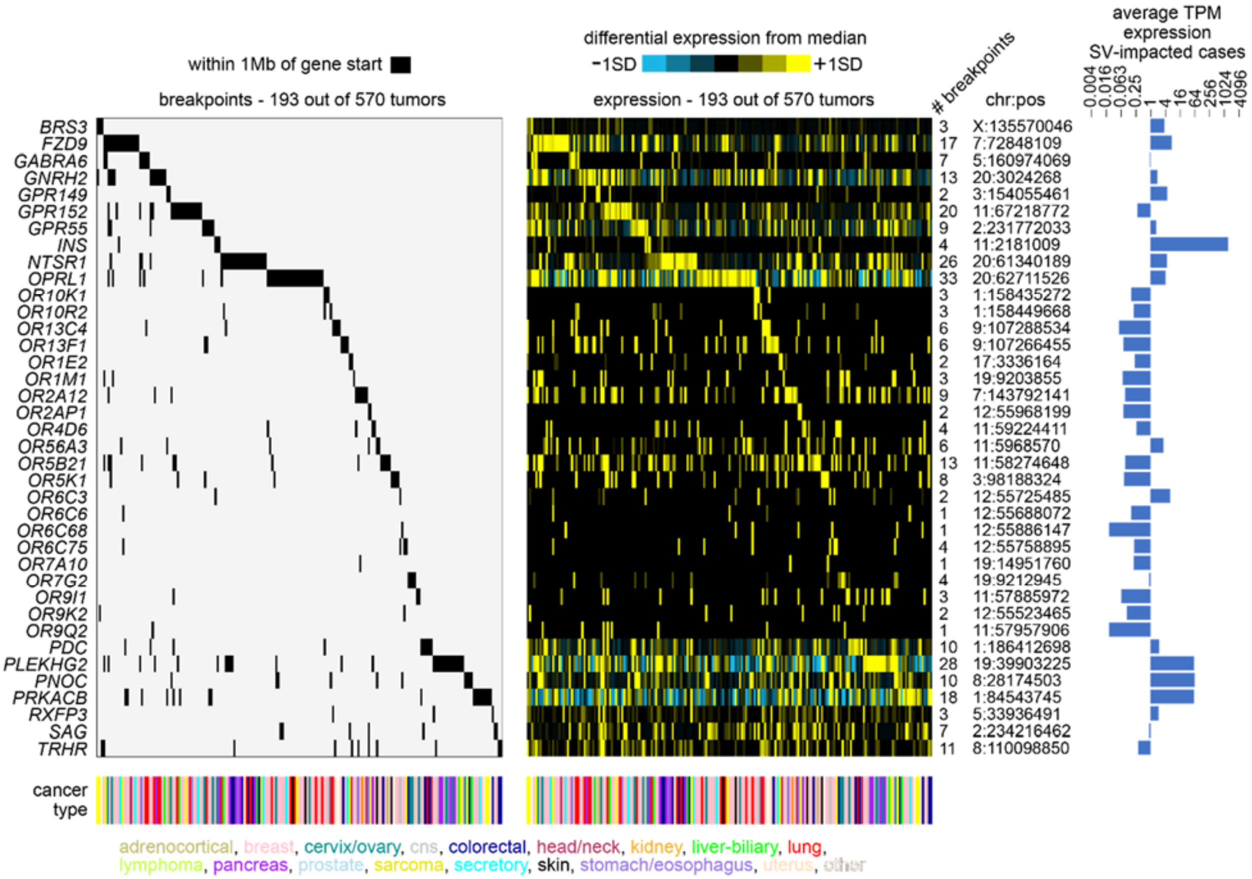

Figure S3. Somatic SV-mediated cis-regulatory alterations involving G protein-coupled receptor signaling pathway genes across the POG570 cohort. Related to Figure 2. Patterns of SV versus expression for genes with GO annotation 'G protein-coupled receptor signaling pathway' and significant SV-expression across the POG570 cohort (FDR<10%, 1 Mb region window, correcting for tumor type and CNA). Tumors represented are those for which an SV breakpoint was within 1Mb of the gene and for which the gene had expression levels 0.4SD greater than the sample median. Off to the right, the numbers of impacted tumors for each gene with nearby SV event combined with over-expression are provided, along with TPM (transcripts per million) of the SV-impacted cases. G protein-coupled receptor genes represent a large gene family with a high degree of homology. Still, the observed SV-associated patterns are not attributable to homology-related technical artifacts (e.g., incorrect mapping of SVs or RNAs due to alignment problems). As observed here, different genes appear altered in different samples. Also, the SV breakpoints involved with these genes occur outside of the gene itself, i.e., within the regulatory regions in proximity to the gene, and for genes in a family with shared homology, such homology would not extend to the gene regulatory regions.

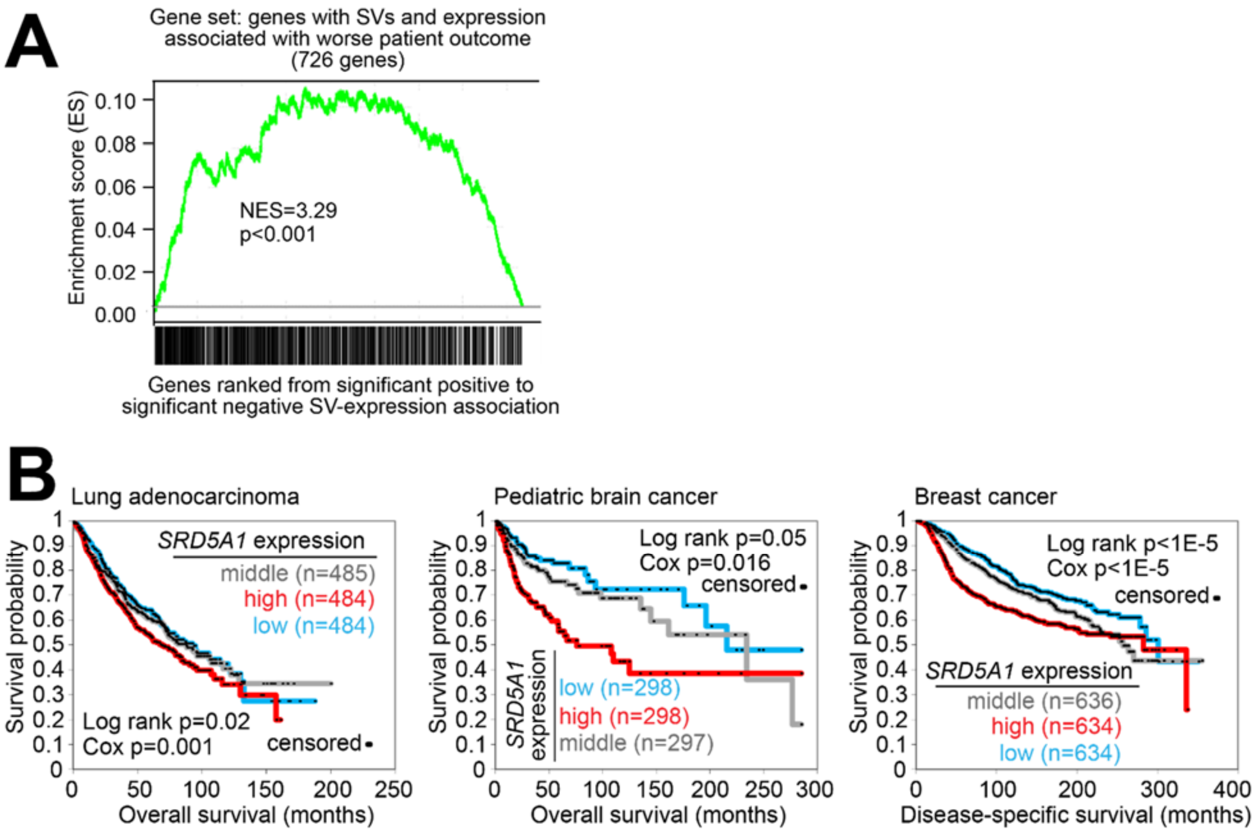

Figure S4. Additional information regarding somatic SV-mediated cis-regulatory alterations in the POG570 cohort and patient survival. Related to Figure 3. (A) Enrichment of positive SV-expression associations with genes associated with worse patient survival by GSEA method. Profiled genes were ranked from significant positive to significant negative SV-expression association (based on ~16700 genes), and the relative positions of 726 genes with both nearby SV breakpoints and expression associated with worse patient outcome (from Figure 3a) were evaluated for enrichment within the positive SV-expression associations by GSEA method (Subramanian et al., 2005). Line graph represents normalized enrichment scores (NES) with associated significance of enrichment. In contrast to main Figure 3A, the significant enrichment association represented here (denoted by the highly positive curve above the x-axis) does not rely upon any cut point for SV-expression association ($p<0.05$ being used in Figure 3A). GSEA is based on version 4.0.3 of the software, with GSEAPreranked feature and classic enrichment statistic. **(B)** Association of *SRD5A1* expression with patient survival across multiple cancer types and three separate datasets: lung adenocarcinoma (n=1453), pediatric brain tumors (n=893), and breast cancer (n=1904). P-values by log-rank test and by univariate Cox, as indicated. For pediatric brain tumor dataset, p-values corrected for histologic type.

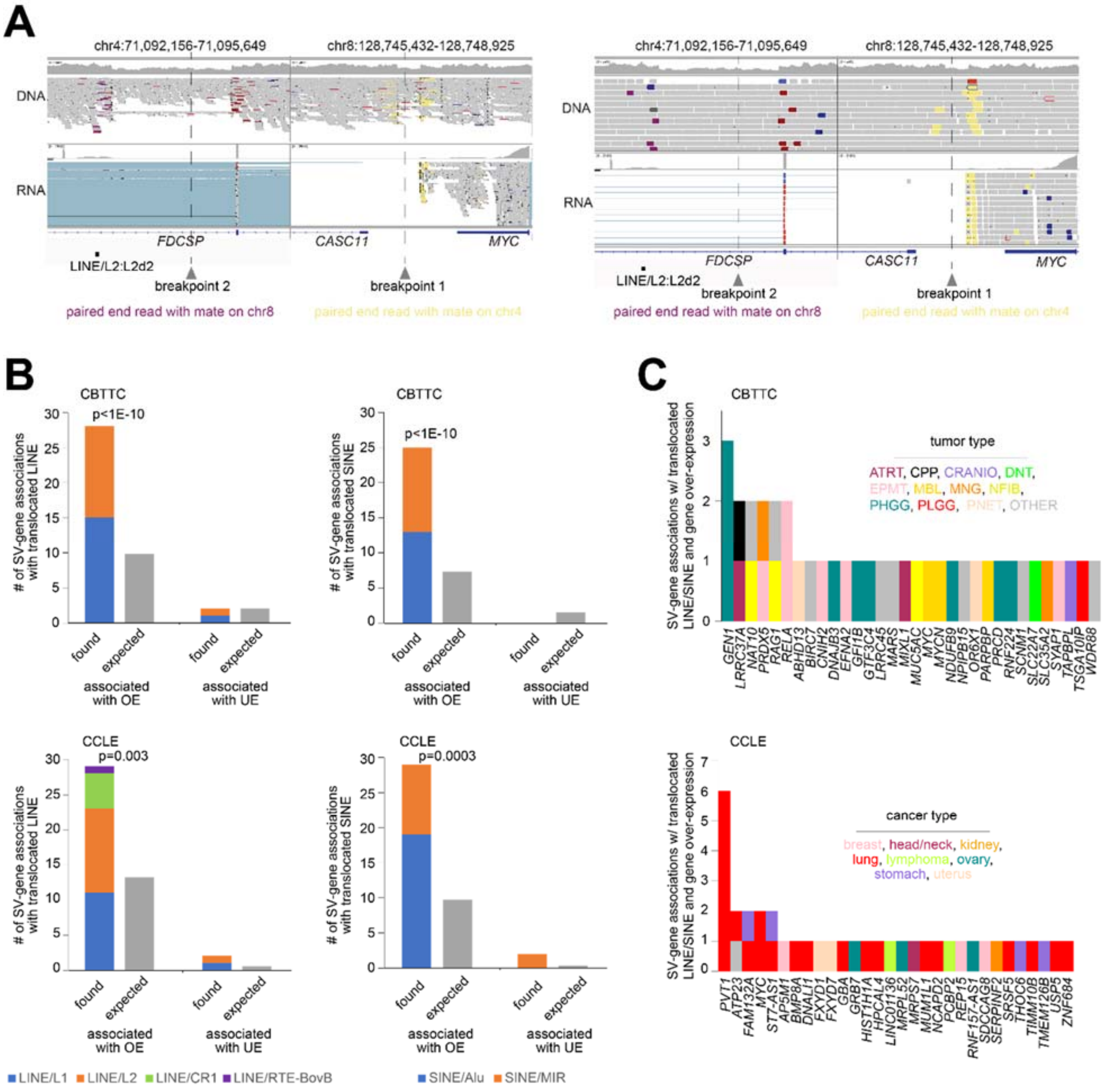

Figure S5. Additional information involving somatic SVs associated with translocated retrotransposons. Related to Figure 4. (A) From POG570 cohort, Integrated Genomics Viewer (IGV) snapshots of a *MYC*-associated SV in patient #32277 involving translocation of a LINE from chromosome 4 to chromosome 8, adjacent to *MYC*. Mapped reads are found to support the SV and associated altered expression for both DNA and RNA datasets. Panel on the right zooms in to examine more closely a subset of reads represented in the left panel. **(B)** For the CBTTC cohort (top) and the CCLE cohort (bottom), numbers of SV breakpoint associations involving the translocation of a LINE or SINE retrotransposon within 20kb of the SV breakpoint in proximity to the gene (and closer than any

enhancer for the unaltered gene), as tabulated for the entire set of SV breakpoint associations with breakpoint mate on the distal side from the gene, as well as for the subsets of SV breakpoint associations involving altered gene expression. OE, over-expression; UE, under-expression. Enrichment p-values by chi-squared test. **(C)** For the CBTTC cohort (top) and the CCLE cohort (bottom), by gene and by cancer type, the number of SV breakpoint associations from part a involving the translocation of LINE or SINE retrotransposons. For parts b-c, SV-associated over-expression or under-expression is defined as FDR<10% for the gene by distance metric method (Zhang et al., 2019) using 1Mb region window, with corrections for tumor type and CNA, and expression > 0.4SD or < -4SD, respectively, from median for the case harboring the breakpoint.

A

therapeutic agent (class I)

subcategory/superset relationship

targeted pathway	anthracyclines	aromatase inhibitor	DNA alkylating	DNA synthesis inhibitor	ER antagonist	HER2 inhibitor	immunotherapy	LHRH agonist	mitotic inhibitor	mTOR inhibitor	taxanes	topoisomerase I inhibitor	topoisomerase II inhibitor	VEGF inhibitor	# patients
AKT-mTOR	12	24	14	19	19	0	0	2	0	25	13	0	0	0	29
cell cycle	7	10	8	5	10	0	0	3	0	1	8	0	0	0	10
DNA damage	153	78	336	200	69	15	13	16	36	11	146	47	30	33	336
DNA synthesis	167	81	281	253	74	17	7	14	31	18	125	69	30	51	343
hormone	93	116	105	78	95	14	3	20	15	21	87	4	2	5	150
immune system	2	2	13	5	2	1	21	0	2	0	7	0	1	0	21
mitotic inhibitor	119	71	173	92	63	18	9	16	37	10	168	5	13	2	196
receptor kinase	28	12	71	73	17	21	3	2	11	3	24	44	6	55	124
# patients	167	116	336	253	95	21	21	20	37	25	168	69	30	55	

B

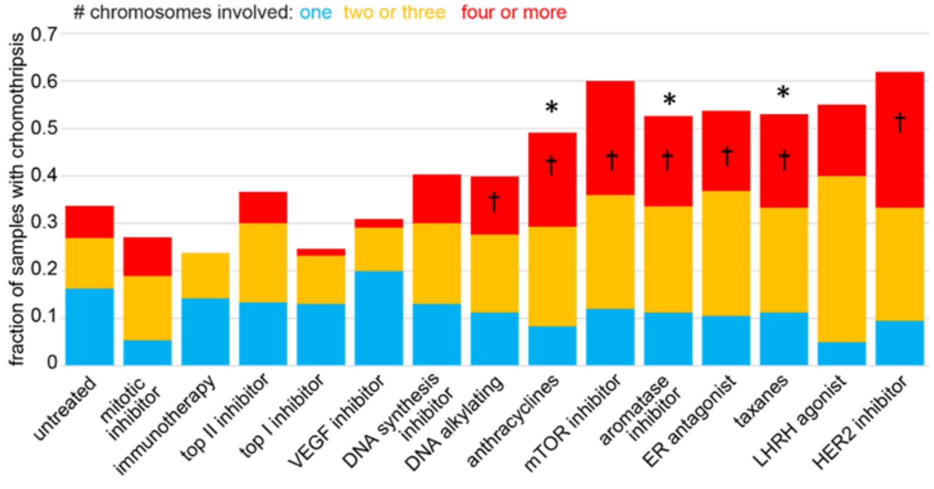

* p<0.001, all chromothripsis cases † p<0.04, 4+ chromosomes

C

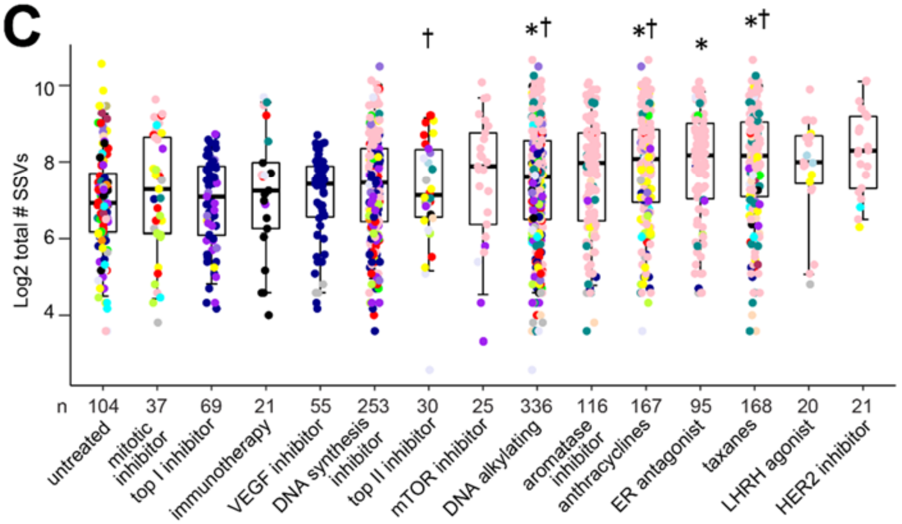

* p<0.0001, uncorrected † p<0.05, corrected for cancer type

cancer type adrenocortical, bladder/kidney, breast, cervix/ovary, cns, colorectal, head/neck, liver-biliary, lung, lymphoma, pancreas, prostate, sarcoma, secretory, skin, stomach/esophagus, uterus, other

Figure S6. Associations of overall SV burden and chromothripsis with patient therapy (by therapeutic agent). Related to Figure 5. **(A)** Patient overlap between major therapy subgroups as defined by therapeutic agent (columns) and by targeted pathway (rows), as represented in the POG570 cohort. Red boxes denote which therapeutic agents are associated with which targeted pathways. All patients in the “DNA damage” targeted pathway group (n=336) were treated with DNA alkylating agents. Most of the patients in the “mitotic inhibitor” targeted pathway group (n=196) were treated with taxanes (n=168). Anthracylines were considered as part of the “DNA synthesis” targeted pathway group, along with DNA synthesis inhibitors and topoisomerase I and II inhibitors. **(B)** Fraction of patients with chromothripsis events, according to patient treatment group (by therapeutic agent). Enrichment p-values (comparing each treatment group with the rest of the tumors) by one-sided Fisher’s exact test. **(C)** Total number of SVs detected in the tumor, according to patient treatment group (by therapeutic agent). P-values (comparing each treatment group with the rest of the tumors) by t-test or regression model incorporating cancer type, as indicated. Boxplot represents 5%, 25%, 50%, 75%, and 95%. Tumors from patients treated with DNA alkylating agents (i.e., DNA damage inducers), taxanes, or DNA alkylating agents, biopsied after therapy, had both a higher representation of chromothripsis events and higher overall numbers of detected somatic SVs.

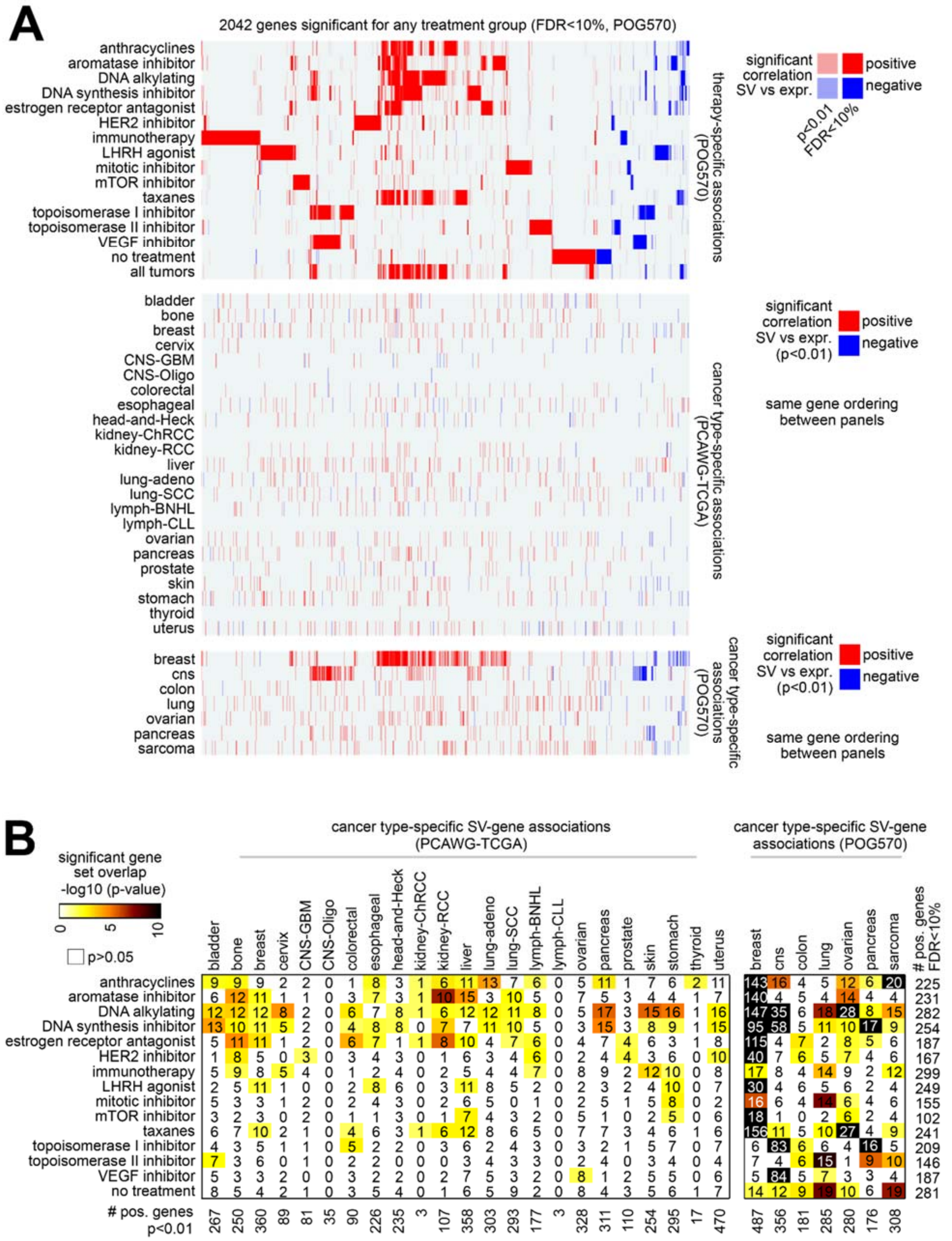
A
2042 genes significant for any treatment group (FDR<10%, POG570)
anthracyclines
aromatase inhibitor
DNA alkylating
DNA synthesis inhibitor
estrogen receptor antagonist
HER2 inhibitor
immunotherapy
LHRH agonist
mitotic inhibitor
mTOR inhibitor
taxanes
topoisomerase I inhibitor
topoisomerase II inhibitor
VEGF inhibitor
no treatment
all tumors
therapy-specific associations (POG570)
significant correlation SV vs expr.
positive
negative
p<0.01
FDR<10%
bladder
bone
breast
cervix
CNS-GBM
CNS-Oligo
colorectal
esophageal
head-and-Heck
kidney-ChRCC
kidney-RCC
liver
lung-adeno
lung-SCC
lymph-BNHL
lymph-CLL
ovarian
pancreas
prostate
skin
stomach
thyroid
uterus
cancer type-specific associations (PCAWG-TCGA)
significant correlation SV vs expr. (p<0.01)
positive
negative
same gene ordering between panels
breast
cns
colon
lung
ovarian
pancreas
sarcoma
cancer type-specific associations (POG570)
significant correlation SV vs expr. (p<0.01)
positive
negative
same gene ordering between panels
B
significant gene set overlap -log10 (p-value)
0
5
10
p>0.05
cancer type-specific SV-gene associations (PCAWG-TCGA)
cancer type-specific SV-gene associations (POG570)
pos. genes FDR<10%
pos. genes p<0.01

Figure S7. Somatic SV-expression associations in POG570 cohort according to therapy are largely distinct from SV-expression associations according to cancer type. Related to Figure 6. (A) Heat map of differential t-statistics by therapy subgroup, evaluating gene expression alterations with nearby SV breakpoint (red, positive correlation with breakpoint; white, not significant), for 1983 genes significant for one or more individual therapy subgroups (FDR<10%). For these POG570 therapy-specific genes, the corresponding cancer types-specific SV-expression association t-statistics in the PCAWG-TCGA cohort (derived previously (Zhang et al., 2019)) and in the POG570 cohort are represented. **(B)** By one-sided Fisher's exact test, significance of overlap between the POG570 therapy-specific associations and the cancer type-specific associations by either PCAWG-TCGA or POG570 (respectively defined using FDR<10% and p<0.05, 1Mb region, correcting for CNA). Between the POG570 therapy and PCAWG-TCGA results sets, no notable significant overlaps between therapy-specific gene associations and cancer type-specific associations, respectively, are observed. Between the POG570 therapy and POG570 gene-level results sets, significant overlaps are observed. The overlaps between the POG570 breast cancer-specific gene set and several therapy-specific gene sets, for example, would reflect the patient tumor overlaps between the two respective sets of subgroups.
